# Improving multibreed genomic prediction for breeds with small populations by modeling heterogeneous genetic (co)variance blockwise accounting for linkage disequilibrium

**DOI:** 10.1186/s40104-025-01303-9

**Published:** 2025-12-20

**Authors:** Weining Li, Siyu Li, Heng Du, Qianqian Huang, Yue Zhuo, Lei Zhou, Jinhua Cheng, Wanying Li, Jicai Jiang, Jianfeng Liu

**Affiliations:** 1 State Key Laboratory of Animal Biotech Breeding, Frontiers Science Center for Molecular Design Breeding (MOE), College of Animal Science and Technology, China Agricultural University, Haidian, Beijing, 100193 China; 2 Institute of Animal Science, Jiangsu Academy of Agricultural Sciences, Nanjing Jiangsu, 210014 China; 3 Department of Animal Science, North Carolina State University, Raleigh North Carolina, 27695 USA

**Keywords:** Heterogeneous genetic (co)variance, Linkage disequilibrium, Multibreed genomic prediction, Multitrait Bayesian model, Small-population breed

## Abstract

**Background:**

Multibreed genomic prediction (MBGP) is crucial for improving prediction accuracy for breeds with small populations, for which limited data are often available. Recent studies have demonstrated that partitioning the genome into nonoverlapping blocks to model heterogeneous genetic (co)variance in multitrait models can achieve higher joint prediction accuracy. However, the block partitioning method, a key factor influencing model performance, has not been extensively explored.

**Results:**

We introduce mbBayesABLD, a novel Bayesian MBGP model that partitions each chromosome into nonoverlapping blocks on the basis of linkage disequilibrium (LD) patterns. In this model, marker effects within each block are assumed to follow normal distributions with block-specific parameters. We employ simulated data as well as empirical datasets from pigs and beans to assess genomic prediction accuracy across different models using cross-validation. The results demonstrate that mbBayesABLD significantly outperforms conventional MBGP models, such as GBLUP and BayesR. For the meat marbling score trait in pigs, compared with GBLUP, which does not account for heterogeneous genetic (co)variance, mbBayesABLD improves the prediction accuracy for the small-population breed Landrace by 15.6%. Furthermore, our findings indicate that a moderate level of similarity in LD patterns between breeds (with an average correlation of 0.6) is sufficient to improve the prediction accuracy of the target breed.

**Conclusions:**

This study presents a novel LD block-based approach for multibreed genomic prediction. Our work provides a practical tool for livestock breeding programs and offers new insights into leveraging genetic diversity across breeds for improved genomic prediction.

**Supplementary Information:**

The online version contains supplementary material available at 10.1186/s40104-025-01303-9.

## Background

Genomic prediction is a method that uses genetic markers across the entire genome to estimate genomic breeding values [1]. Genomic prediction has demonstrated marked effectiveness in animal and plant breeding [2, 3], particularly in international transboundary breeds with large reference populations [4]. However, for breeds with small populations and unique genetic characteristics, the challenge lies in establishing a sufficiently large reference population [5–7]. Additionally, similar difficulties arise in establishing reference populations of sufficient size for traits that are difficult to measure [8, 9]. The prediction accuracy of genomic breeding values cannot be reliably guaranteed when an adequately large reference population is unavailable [10].

To address these issues, an increasing number of studies have leveraged information from multiple breeds for genomic prediction (MBGP) [11–13]. Unlike multi-population joint prediction, different breeds usually do not share common ancestors, making it impossible to conduct joint evaluation through pedigree connections. The reduced cost of genotyping and the emergence of genomic prediction techniques have provided new opportunities for MBGP. The traditional approach to MBGP involves treating different breeds as purebred populations and applying conventional univariate models [14, 15]. However, owing to differences in linkage disequilibrium (LD) patterns and allele frequencies among breeds, simply merging data from different breeds often results in lower accuracy than within-breed genomic prediction [16–18].

The multi-trait model offers a more promising approach for MBGP by treating the same traits in different breeds as distinct but potentially correlated traits of a single breed [19, 20]. This method can flexibly account for the genetic correlations between traits in different breeds. Significant genetic heterogeneity in cholesterol traits across ancestries has been reported, with SNP showing concordant effects more frequently found in certain genomic regions, such as regulatory regions [21]. Additionally, a study on beef cattle revealed differences in the degree of genetic correlation between breeds across different genomic regions [22]. Compared with within-breed prediction, the incorporation of heterogeneous genetic (co)variance in MBGP has been shown to achieve higher accuracy [18], where genome partitioning employs a fixed number of adjacent SNPs. However, the genomic regions defined by this partitioning method may not accurately represent true haplotype segments, potentially affecting the model's predictive performance. In a recent study, a multibreed genomic prediction model that incorporates local genetic correlations was developed [23]. However, this model categorizes the genome into only three types of regions: those with positive, negative, or neutral correlations. By assuming these fixed correlation values across the entire genome, this model potentially limits its overall predictive performance.

In this study, we developed an MBGP BayesA model accounting for blockwise heterogeneous genetic (co)variance based on linkage disequilibrium (mbBayesABLD), which enhances interbreed information sharing by utilizing within-block genetic correlations. Through extensive evaluations using both simulated datasets and real data from animal and plant populations, we demonstrate that compared with conventional joint prediction models, mbBayesABLD consistently achieves higher prediction accuracy.

## Methods

### Datasets

#### Simulation

We simulated two multibreed pig populations with growth rate traits using QMSim (v1.10) software [24]: one with two breeds of different population sizes and another with three breeds of equal population sizes (Fig. S1). Different mating designs and selection criteria were used for individuals from a common historical population, resulting in different LD patterns and allele frequencies (Table 1). For the last three generations of all the breeds, two individuals per litter were randomly selected for further analysis. In the two-breed simulation, 3,000 individuals from breed A and 600 from breed B were simulated. In the three-breed simulation, 600 individuals were simulated for each breed. A total of 50,058 evenly distributed SNPs across the genome were selected for further analysis.

**Table 1:** Parameters in the simulation of genotypes and phenotypes across different breeds

**Scenarios**	**Breed**	**Gen**	**Sel**	***N***_ind_	***h***^**2**^	**Mean**
Two breeds	A	40	phen/h	3,000	0.5	1.0
	B	10	rnd	600	0.3	0.5
Three breeds	A	70	rnd	600	0.5	1.5
	B	40	phen/h	600	0.4	1.0
	C	10	phen/l	600	0.3	0.5

Gen refers to the number of generations during the second phase of breed formation, following the selection of founder populations from historical populations for each breed. During the same phase, Sel refers to the individual selection criteria, where 'phen/h' indicates selecting individuals with high phenotypic value, 'phen/l' indicates selecting individuals with low phenotypic value, rnd means random selection of individuals, *N*_ind_ is the number of individuals used for genomic prediction, and *h*^2^ is the heritability of traits. The mean corresponds to the population mean used in the simulation of the phenotypes

Heterogeneous genetic (co)variance was simulated by generating phenotypes using custom R scripts. SNPs were grouped according to the genome partitioning method proposed in this study. We randomly selected 300 blocks and chose one SNP per block as a QTL, assuming uncorrelated effects. Additionally, 10 blocks were randomly selected, with 10 SNPs per block chosen as QTLs, assuming that the correlation of these QTL effects between breeds is represented by *r*_g_. For *r*_g_, two scenarios were considered: one with a constant *r*_g_mean_ (referred to as ‘identical') and another sampled from a uniform distribution U(−1, 1) and adjusted to *r*_g_mean_ (referred to as ‘uniform'). In the two-breed simulation, *r*_g_mean_ was set to 0.2, 0.5, or 0.8, whereas in the three-breed simulation, *r*_g_mean_ was set to 0.2. The two-breed simulation results indicate that, under our settings, heritability had little effect on prediction accuracy. Phenotypes were simulated by adding fixed breed effects and random residual effects to the additive genetic effects. The entire simulation process was repeated 20 times. Details of the simulation are provided in the Supplemental Methods (Additional file 1).

#### Real data

Using two public datasets, we analyzed four traits from livestock and plants (Table 2). The pig dataset contains two breeds, Yorkshire (YY) and Landrace (LL), with 641 and 228 observations, respectively [25]. The pigs were raised under a consistent feeding environment, provided with the same commercial diets and free access to water, and were slaughtered under standardized conditions. A total of 37,304 markers were retained after quality control procedures. The phenotypic data included two meat quality traits: the marbling score (MS) and the proportion of fat areas in the image (PFAI). The bean dataset consisted of three small-population panels: the newly composed climbing bean panel (VEC), the Andean diversity panel (ADP), and the elite Andean breeding panel (VEF) [26]. The numbers of individuals in the three panels were 344, 357 and 587. Two agronomic traits, 100-seed weight (100SdW) and yield, were used in the bean dataset. The heritability of all the traits was estimated using a single-trait GBLUP model to provide insights into their genetic architecture (Table 2). Detailed descriptions of each dataset are available in the Supplemental Methods (Additional file 1).

**Table 2:** Summary description of the two real datasets

**Dataset**	***N***_SNP_	**Traits**	**Breed**	***N***_ind_	**Phenotype**	**Heritability**
Pig	37,304	PFAI	YY	641	2.02 (1.26)	0.22 (0.07)
			LL	228	2.37 (1.42)	0.31 (0.16)
		MS	YY	641	1.43 (0.48)	0.19 (0.07)
			LL	228	1.57 (0.54)	0.33 (0.16)
Bean	14,913	Yield	VEC	344	2,049.09 (323.09)	0.56 (0.08)
			ADP	357	1,009.93 (172.36)	0.49 (0.05)
			VEF	587	1,089.98 (235.58)	0.64 (0.06)
		100SdW	VEC	344	40.88 (6.20)	0.20 (0.07)
			ADP	352	38.91 (6.08)	0.58 (0.05)
			VEF	562	39.68 (4.66)	0.19 (0.06)

In terms of the analyzed traits, PFAI is the proportion of fat areas in the image, MS is the marbling score and 100SdW is the 100-seed weight of the bean. In the analyzed population, YY represents Yorkshire pigs, LL represents Landrace pigs, VEC represents the newly composed climbing bean panel, ADP represents the Andean diversity panel, and VEF represents the elite Andean breeding panel. *N*_SNP_ represents the number of SNPs used in this study, and *N*_ind_ represents the number of individuals with both phenotype and genotype information. The phenotypes are presented as the mean (standard deviation). Heritability was estimated using a single-trait GBLUP model and is reported as the mean (standard error)

### Genomic structure analysis

Genetic differences between breeds are a key factor influencing combined predictions. Firstly, we generated PCA plots using genotype data by PLINK (v1.90) [27]. Using the LD metric *r*^2^ calculated in PLINK, we computed the correlation coefficients of *r*^2^ for all the SNP pairs within a 10-Mbp window (R10Mbp) to assess the consistency of the LD pattern [28]. Additionally, we calculated the Pearson correlation coefficients between the allele frequencies of all the populations on the basis of the SNP markers that segregated simultaneously in both populations.

### mbBayesABLD model

To model heterogeneous genetic (co)variance across different genomic blocks, we employed a multitrait BayesA model:

$$\begin{array}{c}{{\varvec{y}}}_{l}={\mathbf{X}}_{l}{{\varvec{b}}}_{l}+{\sum }_{i=1}^{s}{\sum }_{j=1}^{{m}_{\text{i}}}{{{\varvec{z}}}_{ijl}a}_{ijl}+{{\varvec{e}}}_{l}\end{array}$$

where ${{\varvec{y}}}_{l}$ is the phenotype vector of breed *l*, with corrected phenotypes in the bean dataset and raw phenotypes in other datasets. Vector ${{\varvec{b}}}_{l}$ is the fixed effect(s) of breed *l* with a uniform prior. For the bean and simulation dataset, only the population mean was considered a fixed effect, while an additional fixed effect for sex was included in the pig dataset. The number *s* is the number of blocks across all chromosomes, ${m}_{i}$ is the number of SNPs in block *i*, ${a}_{ijl}$ is the allelic substitution effect of breed *l* at SNP *j* within block *i*, and ${{\varvec{z}}}_{ijl}$ is a column vector where all values represent the minor allele count (0, 1 and 2 for genotypes AA, Aa and aa, respectively) for SNP *j*. This effect follows a multivariate normal distribution with the prior marker effect in block *i* being $N\left(0, {\mathbf{G}}_{i}\right)$, where ${\mathbf{G}}_{i}$ is the (co)variance matrix of all marker effects within the block, with a prior inverse Wishart distribution $IW\left(df, {\mathbf{B}}_{i}\right)$. In a recent study, we demonstrated that specifying the scale matrix ${\mathbf{B}}_{i}$ of the inverse Wishart (IW) prior distribution from the phenotype can achieve higher prediction accuracy than using noninformative hyperparameters [18]. Therefore, ${\mathbf{B}}_{i}={\widetilde{h}}^{2}\mathbf{P}/[s(df-1){\sum }_{j=1}^{{m}_{i}}2{p}_{j}(1-{p}_{j})]$ was used in this study, where $df=4+p$, *p* is the number of breeds, and ${\widetilde{h}}^{2}$ is the prior of heritability, which was set to 0.5, a moderately informative and neutral value commonly used in practice. **P** is a diagonal matrix with phenotypic variance along the diagonal, ${p}_{j}$ is the allele frequency of SNP *j* in block *i* across breeds, and ***e*** is the residual effect vector that follows $N\left(0, {{\mathbf{I}}_{n}\otimes \mathbf{R}}_{0}\right)$, where *n* is the number of individuals, and ${\mathbf{R}}_{0}\sim IW\left(df,{\mathbf{R}}_{p}\right)$, with ${\mathbf{R}}_{p}={(1-\widetilde{h}}^{2})\mathbf{P}/(df-1)$. Then, the full conditional posterior distributions for ***b***, ***a***, ${\mathbf{R}}_{0}$ and ${\mathbf{G}}_{i}$ were as follows:

$$\begin{array}{c} \textit{P} (\boldsymbol{b|}E{LS}E) \propto N [(\mathbf{X}^{\boldsymbol{\prime}}\mathbf{R}^{-1}\mathbf{X})^{-1} \mathbf{X}^{\boldsymbol{\prime}}\mathbf{R}^{-1}\boldsymbol{y}^{*}, (\mathbf{X}^{\boldsymbol{\prime}}\mathbf{R}^{-1}\mathbf{X})^{-1}] \\ \textit{P}\left(\boldsymbol{a}_{ij} \boldsymbol{|} ELSE\right) \propto N \left[\left(\mathbf{M}^{*^{\boldsymbol{\prime}}}_{i} \mathbf{R}^{-1} \mathbf{M}^{*}_{i} + \mathbf{B}^{-1}_{i}\right)^{-1} \mathbf{M}^{*^{\boldsymbol{\prime}}}_{i} \mathbf{R}^{-1} \boldsymbol{y}^{\dag}, \left(\mathbf{M}^{*^{\boldsymbol{\prime}}}_{i} \mathbf{R}^{-1} \mathbf{M}^{*}_{i} + \mathbf{B}^{-1}_{i}\right)^{-1}\right]\\ \textit{P} (\mathbf{G}_{i} \boldsymbol{|} ELSE) \propto IW \left[df + m_{i}, \left(\sum\limits^{m_{i}}_{i=1} \boldsymbol{a}_{ij} \boldsymbol{a}_{ij}^{\boldsymbol{\prime}} + \mathbf{B}_{i}\right)\right]\\ \textit{P} (\mathbf{R}_{0} \boldsymbol{|} ELSE) \propto IW \left[df + n, \left(\sum\limits^{n}_{i=1} \boldsymbol{e}_{i} \boldsymbol{e}_{i}^{\boldsymbol{\prime}} + \mathbf{R}_{p}\right)\right]\\ \mathbf{R} = \mathbf{I}_{n}{\boldsymbol{\otimes}}\mathbf{R}_{0},\ \boldsymbol{y}^{*} = \boldsymbol{y} - \sum\nolimits^{s}_{i=1} \mathbf{M}^{*}_{j} \boldsymbol{a}_{i}\\ \mathbf{M}^{\ast}_{i} = \mathbf{I}_{p}{\boldsymbol{\otimes}}\mathbf{M}_{i},\ \boldsymbol{y}^{\dag} = \boldsymbol{y} - \mathbf{X}{\boldsymbol{b}} - \sum\nolimits^{s}_{t \neq i} \mathbf{M}^{*}_{t} \boldsymbol{a}_{t}\end{array}$$

where ${\mathbf{M}}_{i}$ is the genotype matrix coded as 0, 1, 2 for the minor allele, and ${{\varvec{y}}}^{*}$ and ${{\varvec{y}}}^{+}$ are vectors of corrected phenotypic values. The missing phenotypes were imputed according to the method proposed by Gianola and Fernando [29]. For more detailed information about the derivation of the posterior distribution, refer to the Supplemental Methods.

**Fig. 1:**
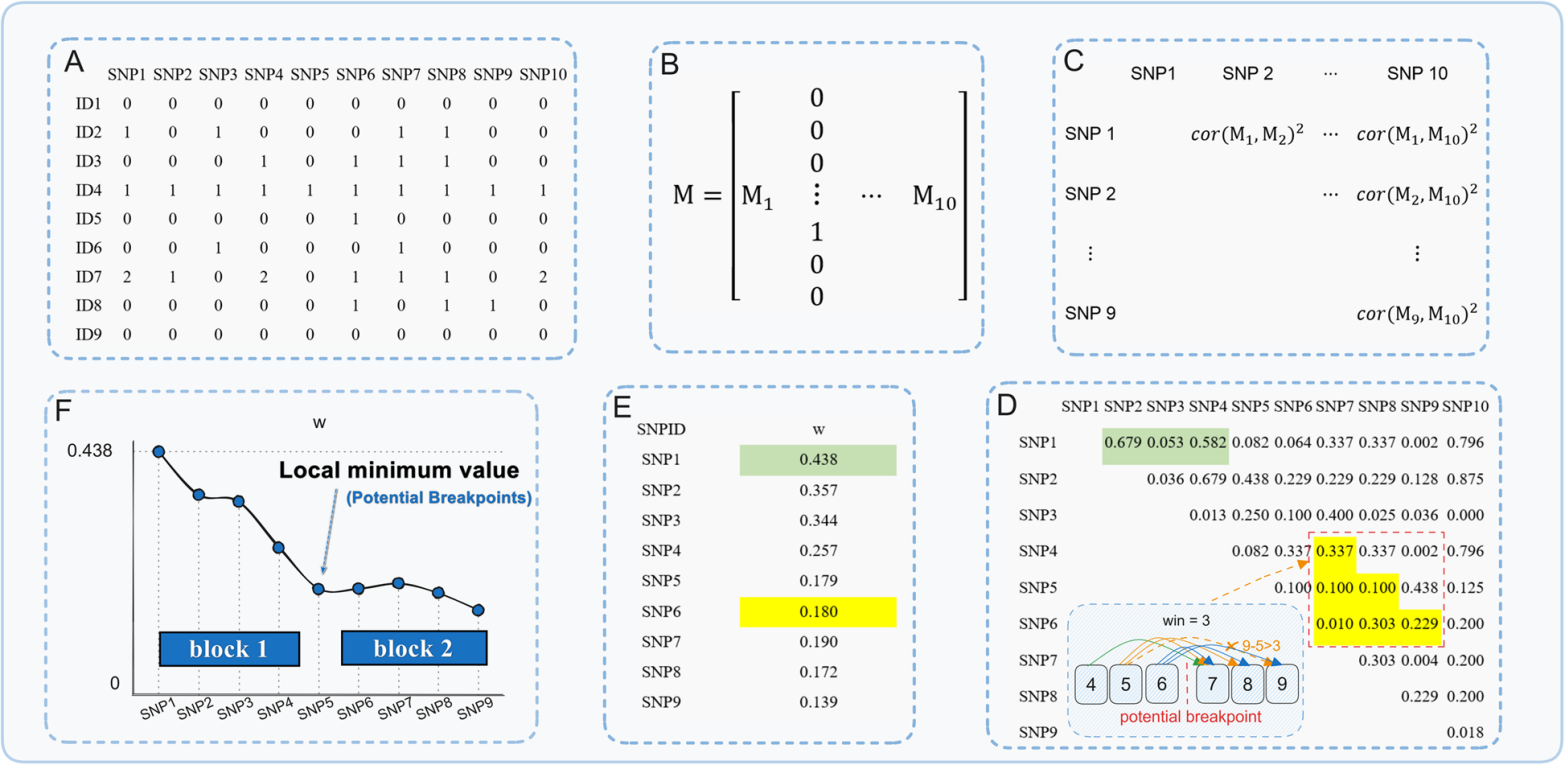
Procedure for genomic block partitioning based on linkage disequilibrium (LD). The values (0, 1, or 2) in the SNP content matrix represent the number of minor alleles in an individual at the given locus, while $Cor\left({SNP}_{i},{SNP}_{j}\right)$ represents the Pearson correlation coefficients of the allele content for markers *i* and *j*. *w* represents the average correlation coefficient between all SNP pairs on both sides of a given SNP, where the distance between the pairs falls within the window (*win*), assuming that this SNP serves as the genomic partition breakpoint. This value reflects the degree of linkage disequilibrium between SNPs on both sides of the specified SNP

A key feature of our model is the application of a novel genome block partitioning method when heterogeneous genetic (co)variance is incorporated. First, we calculated a statistic ${w}_{k}$ based on the LD to determine whether a given position is suitable as a breakpoint:

$${w}_{k}=\frac{1}{{m}_{k}}\sum {\text{Corr}[{\mathbf{M}}_{i}, {\mathbf{M}}_{j}]}^{2}$$

where ${w}_{k}$ ($k=1,\cdots ,m$) is the statistic *w* of SNP *k* and *m* is the number of SNPs on the chromosome that need to be divided into blocks. ${\mathbf{M}}_{i}$ ($i=max\{1, k-{N}_{win}+1\},\cdots ,k)$ is column *i* of the genotype matrix **M**, coded as 0, 1 or 2 for genotypes A_1_A_1_, A_1_A_2_ and A_2_A_2_, respectively. ${\mathbf{M}}_{j}$ is similar to ${\mathbf{M}}_{i}$ and $j=k+1,\cdots ,min\{i+{N}_{\text{win}},m\}$. $\text{Corr}[{\mathbf{M}}_{i}, {\mathbf{M}}_{j}]$ is the Pearson correlation coefficient between ${\mathbf{M}}_{i}$ and ${\mathbf{M}}_{j}$, where *N*_win_ is a parameter used to control the SNP farthest from *k* that needs to be considered when the correlation coefficient is calculated. In the simulation study, we found that setting *N*_win_ to 50 yielded a relatively optimal prediction accuracy (Fig. S2). ${m}_{k}$ is the number of all vector pairs that match the value range of *i* and *j*. We provide a small example illustrating the detailed calculation process for ${w}_{k}$ in Fig. 1.

Theoretically, the metric ${w}_{k}$ ranges from 0 to 1, with higher values indicating stronger linkages between SNP *k* and its flanking SNPs on both sides. After obtaining the ***w*** vector for a chromosome, we applied the *smooth.spline* function provided by R's stats package to achieve curve smoothing [30]. On the basis of LD calculations and visualization using real genotype data, we found that setting the smoothing parameter *spar* to 0.2 resulted in moderate smoothing of the ***w*** vector and facilitated the identification of LD block breakpoints (Fig. S3). We then searched the entire chromosome for local minima, using these as breakpoints for block delineation. Thus, regional partitioning utilized combined genotype information from all the breeds in the reference population.

### Models used for comparative analysis

In previous studies, we compared the accuracy of within-breed and multibreed genomic prediction models [18]. Building on those findings, this study focuses specifically on multibreed approaches and investigates the advantages of the mbBayesABLD model over other multibreed genomic prediction methods.

#### Bayesian models

In addition to mbBayesABLD with our proposed block partitioning method, two additional strategies were implemented to segment chromosomes in the mbBayesAB framework. First, the haplotype definition method (https://github.com/cadeleeuw/lava-partitioning) was applied to segment the chromosomes [31], with a minimum requirement of 100 SNPs per block. This method also uses LD information to determine breakpoints but emphasizes maintaining consistent block lengths. We refer to this model as mbBayesAB-lava in the subsequent sections. Additionally, a simpler strategy based on a fixed number of SNPs (mbBayesAB-fix) was used for comparison. The number of SNPs in this strategy was set to 100, as this configuration demonstrated optimal performance in previous studies [32, 33]. The comparative analysis also included BayesR [34], a model that accounts for variance heterogeneity by assuming that SNP effects follow a mixture of four normal distributions. The single-trait BayesR model was applied to the MBGP using a reference population created by merging multiple breeds, implemented with the publicly available BayesR software (https://github.com/syntheke/bayesR) under default settings.

The three models (mbBayesAB-fix, mbBayesAB-lava, and mbBayesABLD) accounting for heterogeneous genetic (co)variances in this study were implemented and estimated using our in-house software package mbBayesABLD, which is freely available for download and use from our GitHub repository: https://github.com/CAU-TeamLiuJF/MBGP_CV/tree/main/mbBayesABLD. Analysis of the posterior samples indicated that increasing the number of iterations beyond 30,000 led to highly consistent outcomes across the Bayesian models (Table S1). Therefore, in the analysis of the Bayesian models, the Gibbs sampler ran for 30,000 cycles, with the first 20,000 cycles treated as burn-in and a thinning interval of ten cycles. The same iteration settings were also used in the BayesR analysis.

#### GBLUP models

Two GBLUP models were applied for MBGP: STGBLUP and MTGBLUP. The general model was defined as:

$$\begin{array}{c}\boldsymbol {y}{=}\boldsymbol {X}\boldsymbol {b}{+}\boldsymbol {Z}\boldsymbol {a}{+}\boldsymbol {e}\end{array}$$

where ***a*** is the vector of genomic breeding values, and the other terms are specified as in mbBayesABLD. In STGBLUP, all breeds in the reference population were treated as a single population with a shared genetic background, and single-trait GBLUP was applied, assuming ***a*** ~ $N(0, \mathbf{G}{\sigma }_{a}^{2})$, where **G** is the genomic relationship matrix (GRM) and ${\sigma }_{a}^{2}$ is the additive genetic variance. In MTGBLUP, the same trait measured across breeds was modeled as distinct traits, with ***a*** ~ $N\left(0,{\mathbf{G}}_{0}\otimes \mathbf{G}\right)$, where ${\mathbf{G}}_{0}$ is the (co)variance matrix of genomic breeding values between traits. The GRM was constructed following the method of VanRaden [35] implemented in GMAT software (https://github.com/chaoning/GMAT), using allele frequencies from the joint reference population [36, 37]. Variance components and GEBVs were estimated with DMUAI program of the DMU software [38].

### Cross-validation and model performance assessment

The same MBGP models were applied to both the simulated and real datasets to enable comparative performance evaluation across different data structures (Fig. S4). Five-fold cross-validation (CV) was employed to assess the accuracy and unbiasedness of the GEBVs. The CV process was repeated five times in the simulation and ten times in the real datasets. The same validation subsets were applied across all the models with the reference population formed by merging the respective breeds (Table S2). Prediction accuracy and unbiasedness were assessed by calculating the correlation and regression coefficients between the GEBVs and the pre-adjusted phenotypes (or true breeding values in simulations) for the validation individuals. Refer to the Supplemental Methods (Additional file 1) for details on the CV procedure. Paired *t*-test was performed to assess the statistical significance of mean differences. The computational efficiency of the different models was compared by measuring the runtime and average peak memory consumption for all MBGP models included in the simulation study.

## Results

### Effects of genome segmenting on multibreed prediction accuracy

For MBGP of PFAI, MS, and yield traits, the global genetic correlation estimates from the Bayesian model were significantly lower than those from the MTGBLUP (Tables S3 and S4). Notably, the mbBayesABLD model achieved a significant improvement in prediction accuracy compared with the MTGBLUP model in the analysis of these traits. The estimates of local genetic correlations indicate that genetic correlations vary significantly across different genomic blocks (Fig. S5). We visualized the breakpoint definitions used in the mbBayesABLD and mbBayesAB-lava models (Fig. S6). When an extremely low local *w* value was obtained, the correlation between the SNP on either side of the position was considered weak, suggesting a potential breakpoint between the two haplotype fragments. A comparison of the breakpoints identified by mbBayesABLD and mbBayesAB-lava revealed that those defined by mbBayesABLD typically appeared at positions where local LD trends changed. Some breakpoints identified by mbBayesAB-lava were similar to those in mbBayesABLD, whereas others appeared at the rising or falling points of the curve. Notably, we found that using the merged population as the reference panel yielded the highest prediction accuracy when block partitioning was conducted.

### Simulation datasets

#### Software runtime and memory usage

Simulated data were used to evaluate the computational speed (Table S5). All the models were run using a single core. For joint prediction for two breeds, the computational speed of the multitrait Bayesian models (mbBayesAB-fix, mbBayesAB-lava, and mbBayesABLD) was comparable to that of the MTGBLUP model. Notably, when the reference population included all three breeds, the runtime of the MTGBLUP model was significantly longer than that of the multitrait Bayesian models. Additionally, the three block partitioning strategies did not result in significant differences in model runtimes. In terms of memory usage, BayesR has the highest memory efficiency among all the models (Table S6). When the reference population consists of 2,880 individuals, the peak memory usage of mbBayesABLD during runtime is approximately 2.14 GB, which is slightly greater than that of MTGBLUP (1.54 GB). Different genomic block partitioning strategies have little effect on the memory usage of the multitrait Bayesian models.

#### Combining a large-population breed and a small-population breed

With respect to breeds with small populations, we hypothesized that the prediction accuracy would improve when these breeds were combined with international transboundary breeds with larger populations. Thus, our study examined the performance of mbBayesABLD when it was applied to two breeds with different population sizes (3,000 and 600). Principal component analysis (PCA) revealed distinct genetic clustering between the simulated breeds that closely matched patterns observed in the empirical pig data (Fig. S7), demonstrating the reliability of our genotype simulation. However, the consistency of the LD pattern (Fig. S8) indicated that the LD pattern was conserved across breeds among SNP alleles at short distances (< 500 kbp).

**Fig. 2:**
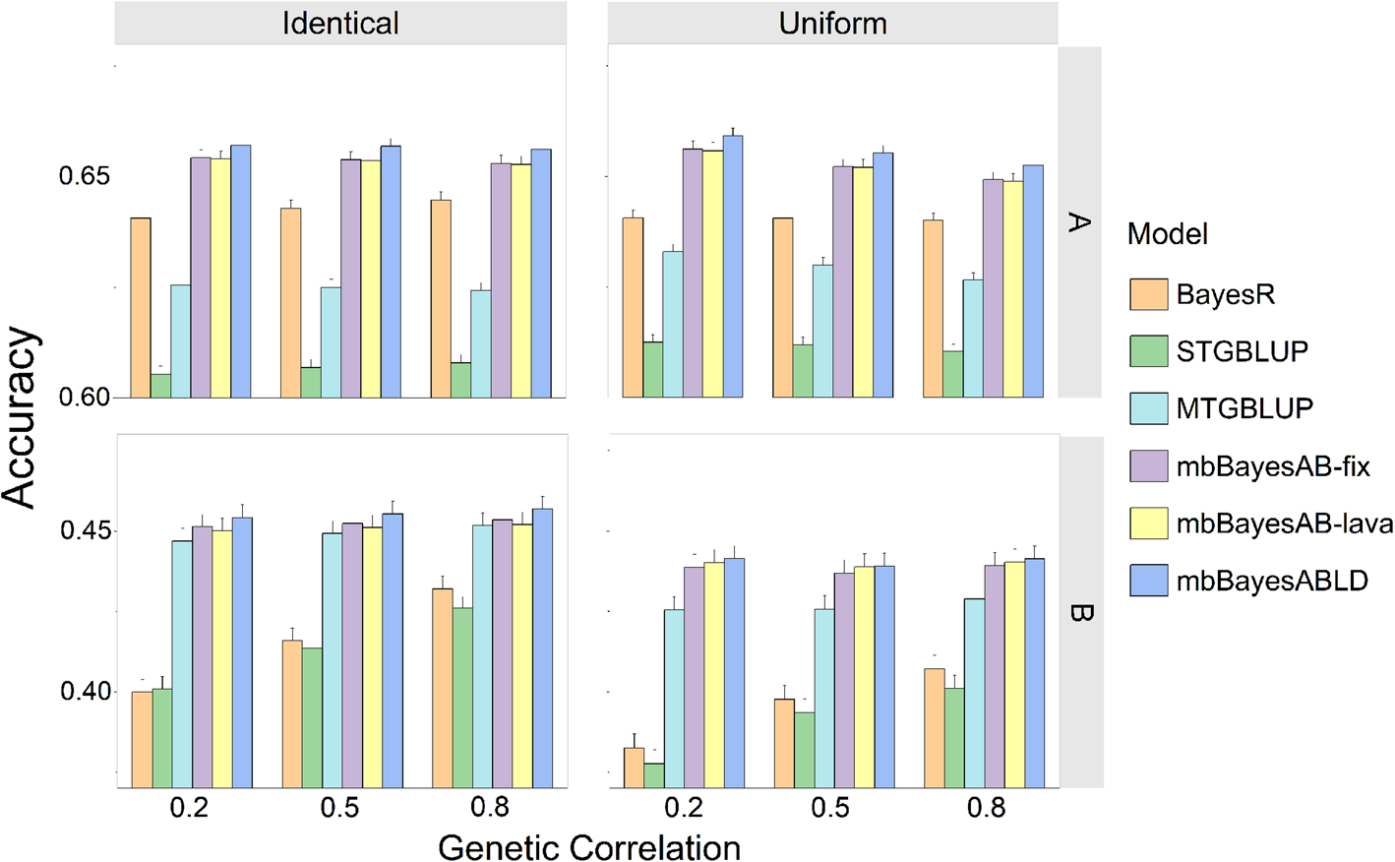
Predictive accuracies of different models using two breeds simulation dataset. The reference for the models included individuals from both breed **A** and **B**. For the genetic correlations, we selected 10 genomic blocks with correlated marker effects across breeds. We designed two scenarios: in the ‘identical’ scenario, correlations are fixed within blocks; in the ‘uniform’ scenario, correlations are sampled from a U(−1, 1) distribution and adjusted to a specified mean. Three different average genetic correlation levels (0.2, 0.5, and 0.8) were set between breeds. The error bars represent the standard errors

As expected, pooling data from multiple breeds and performing genomic prediction using a single-trait animal model significantly reduced the prediction accuracy for the target breed (*P* < 0.05) (Table S7). In all scenarios, MTGBLUP consistently outperformed STGBLUP in terms of prediction accuracy (Fig. 2). When the genetic correlation between breeds was 0.8, STGBLUP achieved a prediction accuracy of 0.61 for breed A, which represented the larger population. In contrast, the mbBayesABLD model attained a higher accuracy of 0.66, corresponding to a relative improvement of approximately 8.2%. In the same scenario, the prediction accuracy for breed B with a smaller population size increased by 1.3%, indicating that joint prediction with mbBayesABLD benefited both breeds. Although the BayesR model outperformed STGBLUP in prediction accuracy, it consistently showed lower accuracy than the three multi-trait Bayesian models. Across all scenarios, the mbBayesABLD model consistently achieved the highest prediction accuracy for breed B. The method of setting genetic correlations across different blocks (identical or uniform) did not significantly impact the ranking prediction accuracy trends of the models, with mbBayesABLD consistently achieving the highest accuracy. Moreover, model performance remained largely unchanged, even when the global genetic correlation between breeds varied.

#### Combining multiple small-population breeds

**Fig. 3:**
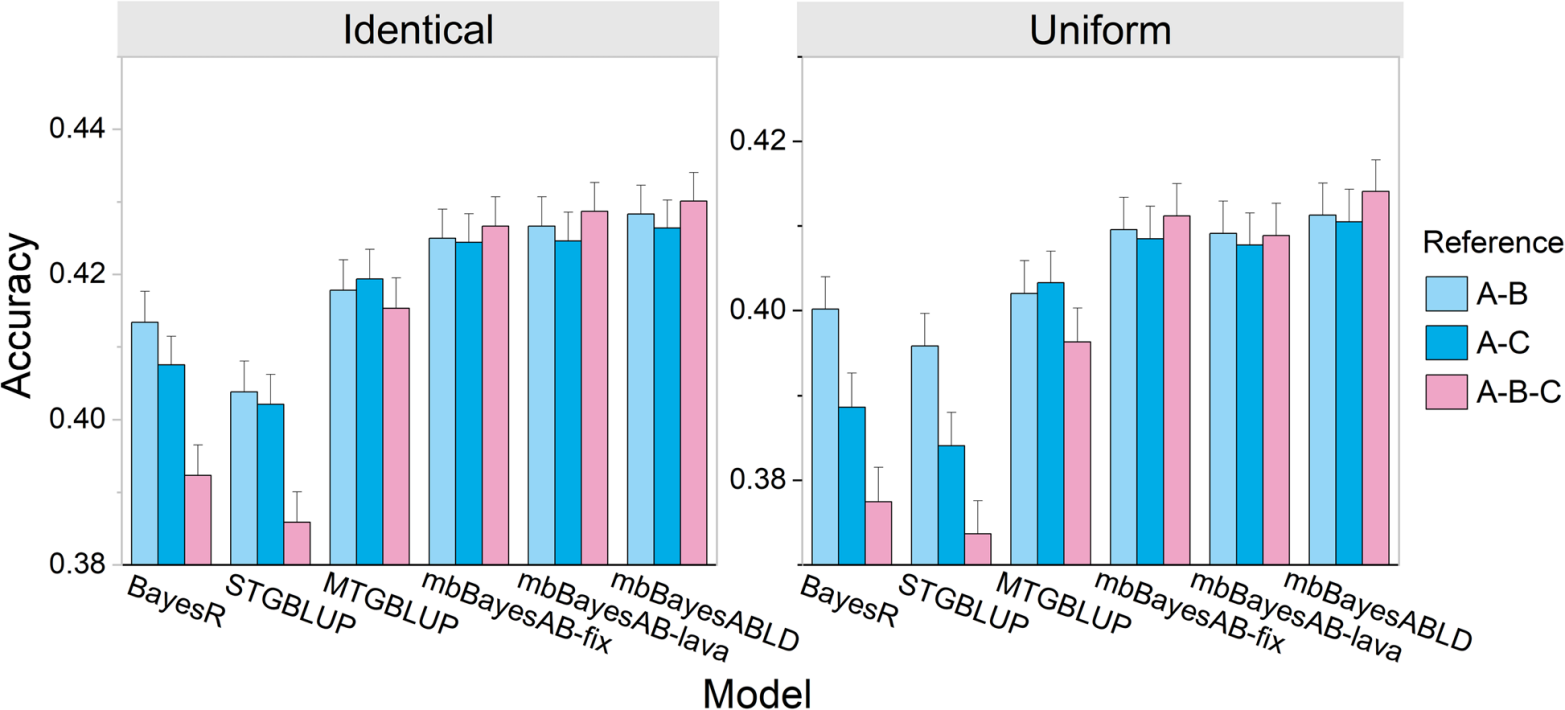
Predictive accuracies of different models for breed A using different reference populations. For example, A-B indicates individuals from both breeds A and B were included in the reference population. In all cases, the validation set consisted solely of individuals from breed A. For genetic correlations, we selected 10 genomic blocks with correlated marker effects across breeds. Two scenarios were explored: in the ‘identical’ scenario, correlations are fixed (0.2) within blocks; in the ‘uniform’ scenario, correlations are sampled from a U(−1, 1) distribution and adjusted to a specified mean (0.2). The error bars represent the standard errors

In this context, we investigated whether the inclusion of breed B and breed C contributes to the prediction of the breeding value of breed A. The results indicated that mbBayesABLD consistently achieved the highest prediction accuracy (Fig. 3). Compared with incorporating breed C, incorporating breed B into the reference population proved more beneficial for breed A. This aligns with expectations, as we partitioned regions using the genotypic combinations of A and B when simulating phenotypes. When the multitrait Bayesian model was applied, compared with including only one or two breeds in the reference, merging three breeds resulted in higher prediction accuracy for breed A. Across all reference population combinations, the BayesR model achieved higher prediction accuracy than STGBLUP, but its accuracy remained lower than those of MTGBLUP and mbBayesABLD. When the local genetic correlations between breeds were equal (identical), mbBayesABLD (0.43) achieved a 10% improvement in prediction accuracy compared with STGBLUP (0.39) when three breeds were merged. It is noteworthy that the lowest prediction accuracy was observed for STGBLUP when all available breeds were combined. In terms of prediction bias, the GBLUP model showed an inflation phenomenon, whereas the Bayesian models generally showed shrinkage (Table S8). On average, compared with the Bayesian models, the GBLUP models exhibited lower bias in MBGP analysis.

### Public pig and bean data

#### Pig data of two breeds with different population sizes

**Fig. 4:**
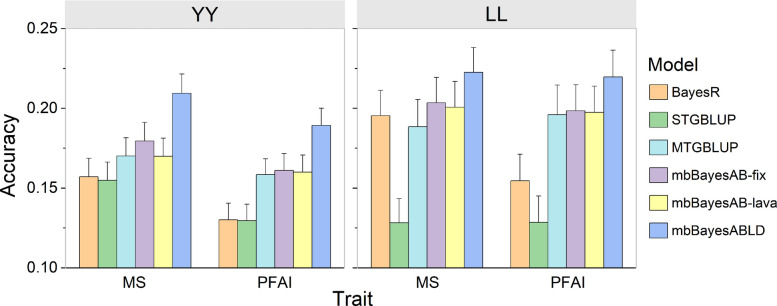
Predictive accuracies of different models using real pig data. The dataset included two pig breeds, Yorkshire (YY) and Landrace (LL). The traits analyzed were marbling score (MS) and proportion of fat areas in the image (PFAI). The reference populations of the models included individuals from both YY and LL. The error bars represent the standard errors

We analyzed the collected pig dataset corresponding to the two breed simulation scenarios. The results showed that LL, a breed with a small population size, benefited more from the mbBayesABLD model than from MTGBLUP and BayesR, which is consistent with our simulation tests (Fig. 4). Additionally, compared with MTGBLUP, mbBayesABLD improved the prediction accuracies for the MS (0.19 vs. 0.22) and PFAI (0.19 vs. 0.22) traits of LL by 15.6% and 9.5%, respectively. Furthermore, the prediction accuracy of mbBayesABLD was significantly greater than that of mbBayesAB-fix and mbBayesAB-lava. In the multitrait MBGP models, mbBayesABLD achieved the highest prediction accuracy in all scenarios. With respect to the MS trait of breed YY, mbBayesABLD (0.21) achieved a 23.5% improvement in accuracy compared with mbBayesAB-lava (0.17), highlighting the critical importance of block partitioning strategies in fitting the heterogeneous genetic (co)variance in MBGP. When GBLUP was applied in the MBGP, the results of the pig data analysis were similar to those of the simulation study. Specifically, STGBLUP significantly decreased the prediction accuracy for the target breed (*P* < 0.05) (Table S9), whereas MTGBLUP outperformed STGBLUP (Fig. 4). Notably, compared with the other models, the mbBayesABLD model exhibited lower prediction bias for LL (Table S10).

#### Bean data with multiple small-population panels

As observed in the simulation, the consistency of the global LD pattern differed only slightly between breeds, with values ranging from 0.55 to 0.74 (Table S9). The genetic distances between different bean panels were smaller than those between pig breeds. However, there were significant differences in allele frequencies between the panels, with ADP showing a negative correlation with the other two bean panels.

**Fig. 5:**
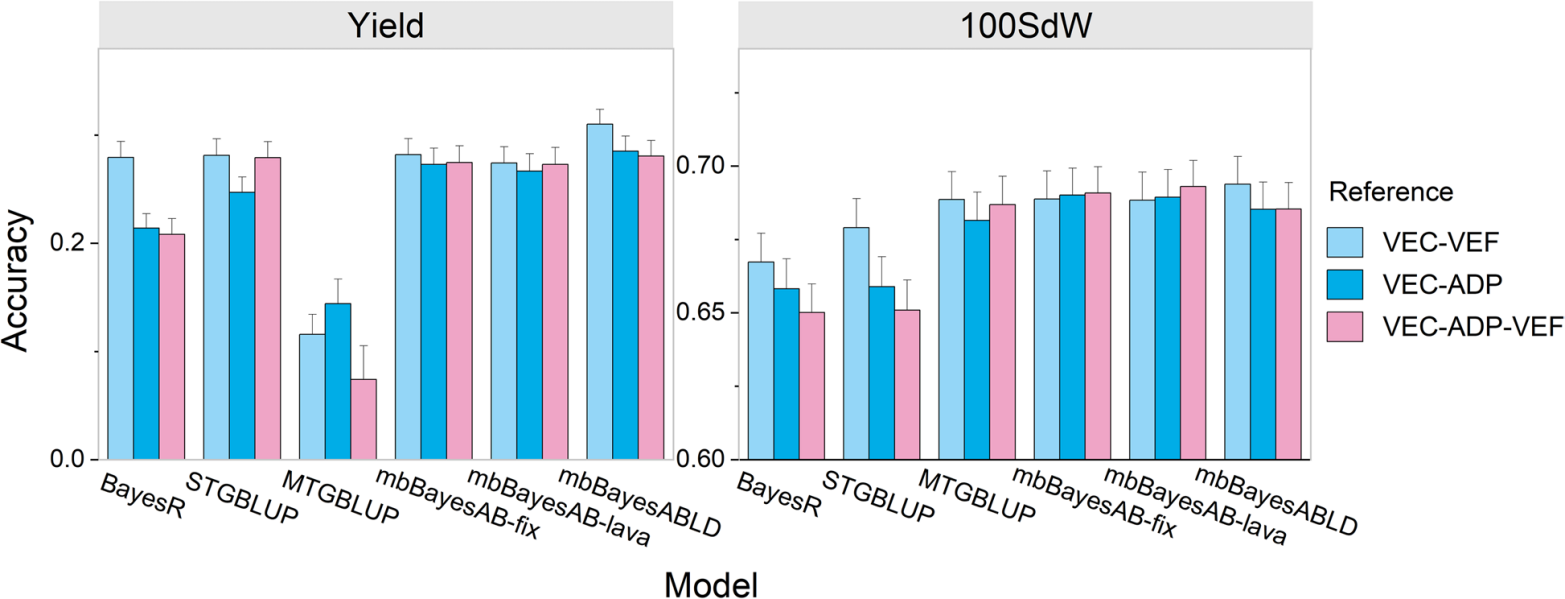
Predictive accuracies of different models for panel VEC using different reference populations. The dataset included three panels: the newly composed climbing bean panel (VEC), the Andean diversity panel (ADP), and the elite Andean breeding panel (VEF). VEC-VEF denotes that individuals from panels VEC and VEF were included in the reference population. In all cases, the individuals used for validation were exclusively from VEC. The analyzed traits included 100-seed weight (100SdW) and Yield. The error bars represent the standard errors

Consistent with the simulation study, we aimed to improve the prediction accuracy of VEC by incorporating data from the other two panels. For the yield and 100SdW traits, merging the VEC and VEF panels into the reference population and applying the mbBayesABLD model achieved the highest prediction accuracy (Fig. 5). Compared with STGBLUP, mbBayesABLD improved the prediction accuracy for the yield and 100SdW traits by 10.3% and 2%, respectively. When VEC and ADP were combined, the prediction accuracy of STGBLUP was 0.21, whereas mbBayesABLD improved the prediction accuracy to 0.29. MTGBLUP outperformed both STGBLUP and BayesR in predicting the 100SdW trait, whereas for yield traits, its predictive accuracy was lower than that of the other two methods. Unexpectedly, the results indicated that incorporating all the panels together did not result in the highest prediction accuracy. For the trait 100SdW, the STGBLUP results displayed a pattern consistent with the simulation data, where including all panels together led to the lowest prediction accuracy.

## Discussion

In this study, we proposed the joint prediction model mbBayesABLD, which enables different breeds to share information more accurately in genome blocks divided on the basis of LD information. Compared with other joint prediction models, mbBayesABLD achieved superior prediction accuracy in both simulation and real data studies. Moreover, our results indicated that mbBayesABLD improved the prediction accuracy for breeds with small populations in multibreed genomic prediction without compromising accuracy for other breeds.

When GBLUP was applied for joint prediction, the multitrait model significantly outperformed the single-trait model in most cases, which is consistent with the findings of previous studies [17, 39]. However, the prediction accuracy did not significantly improve compared with the within-breed predictions. In most cases, the multitrait Bayesian models that account for heterogeneous genetic (co)variance consistently achieved higher prediction accuracy than the other MGBP models did. With mbBayesABLD, the prediction accuracy improved by 21.3% for YY and 15.1% for LL compared with MTGBLUP. With respect to the 100SdW of the bean data, the improvement was even more obvious, with the prediction accuracy increasing by 146%. Our findings strongly suggest that information sharing should be based on the magnitude of genetic correlations among breeds at different genomic positions in joint prediction.

The global genetic correlations between breeds estimated by Bayesian models were significantly lower than those obtained using GBLUP for the analysis of several traits (Table S3 and Table S4). In such cases, multitrait Bayesian models often demonstrated higher prediction accuracy than MTGBLUP did. This could be attributed to MTGBLUP’s tendency to overestimate global genetic correlations between breeds, thereby reducing its predictive performance. Our findings revealed that YY and LL exhibited negative global genetic correlations for the PFAI and MS traits. While no studies have reported on the genetic correlation between YY and LL for MS and PFAI to date, these two breeds are commonly used as maternal lines in production, which suggests that they may have positive genetic correlations. Therefore, further research involving larger populations is necessary to explore the genetic correlations between these two breeds. Additionally, we found that different block partitioning methods play a crucial role in incorporating heterogeneous genetic (co)variances. Our results demonstrated that mbBayesABLD yielded higher prediction accuracy than mbBayesAB-fix and mbBayesAB-lava did in both simulation and real data analysis. In the mbBayesAB-lava method, the emphasis on uniform block size, similar to mbBayesAB-fix, causes the breakpoints to deviate from the local LD trend changes (Fig. S6). This results in segmentations that significantly differ from the actual haplotype segments within the population [40], leading to the assembly of multiple haplotype fragments. Consequently, the accuracy of genetic correlation estimates at that genomic position was affected, thereby limiting the improvement in model prediction accuracy.

The correlation in allele frequencies is also a key factor affecting the performance of joint prediction [13]. In the bean dataset, the correlation for minor allele frequency between VEC and VEF was 0.02, whereas ADP was negatively correlated with the other two panels. This could explain why the best prediction accuracy was observed when the VEC and VEF panels were combined. Joint prediction involves the merging of multiple breeds with different breeding histories, and the resulting changes in allele frequencies can significantly affect the accuracy of marker effect estimates. Additionally, adding ADP to the joint reference population reduced the prediction accuracy for VEC. Therefore, if a breed shows a negative allele frequency correlation with the target breed, its inclusion in the reference population is not recommended, as it may result in suboptimal prediction outcomes.

Several studies have demonstrated that the low consistency of the LD pattern between SNPs and causative variants across populations can negatively impact genomic prediction when a combined population is used [14, 41, 42]. Compared with allele frequency correlations, the consistency of the LD pattern among breeds was more stable across the two real datasets (Table S11). The Landrace and Yorkshire breeds were previously reported to exhibit an LD pattern correlation ≥ 0.80 for distances up to 0.1 Mbp [43], which is slightly greater than the 0.73 estimated in this study. Our results revealed that directly merging multiple breeds led to lower prediction accuracy. Thus, we do not recommend the use of simple merging of multiple breeds for joint prediction when the consistency of the LD pattern between breeds is lower than 0.8. However, our study demonstrated that an average consistency in the LD pattern of 0.6 was sufficient to improve the prediction accuracy of the target breed when using the mbBayesABLD for MBGP. Notably, the optimal values of the LD window size (*N*_win_) and smoothing parameter (*spar*) were identified through systematic sensitivity analyses in this study. As these results were derived using a 50K-density SNP chip array, additional evaluation may be necessary to optimize these parameters under different SNP densities.

In previous works, researchers have discussed the impact of consistency in LD among breeds on MBGP [42, 44], but a method considering LD consistency between breeds has not yet been put into practice. Our study can easily incorporate this information by using the correlation coefficient *w* to measure LD pattern consistency across breeds. However, our study has several limitations. Notably, when the number of breeds exceeds two, adjustments to the model are necessary to handle a more general case. In addition, we assigned a prior covariance value of zero to the marker effects between breeds in this study. In cases where empirical information is reliable, researchers may consider setting informative prior values in the model [45]. We can explore the option of using LD consistency or other information to quantify genetic correlations between breeds and to set nonzero covariance prior values. Although mbBayesABLD was validated only across a maximum of three breeds, the method is intrinsically applicable to analyses involving a substantially larger number of breeds without modification. However, the required computational resources, especially memory, increase rapidly with each additional breed. Conventional GBLUP implementations are expected to encounter even greater convergence challenges than mbBayesABLD when reference populations exceed three breeds. Consequently, analyses encompassing dozens of breeds may require weighted-integration strategies similar to those employed in multinational dairy cattle evaluations to balance computational feasibility and prediction accuracy [45].

## Conclusion

There is increasing interest in breeds with small populations characterized by specific favorable traits, highlighting the importance of developing novel approaches for integrated multibreed prediction using all available genotypic and phenotypic information. This study proposes a new genomic block partitioning strategy, which is applied to fit heterogeneous genetic (co)variances in genomic prediction models. Analyses using both simulated and real datasets demonstrate that the proposed mbBayesABLD model achieves higher prediction accuracy than existing multibreed genomic prediction methods do. This work provides a practical tool for improving multibreed genomic prediction and offers new insights for joint prediction across genetically diverse populations.

## Supplementary Information

Additional file 1: Fig. S1. Schematic representation of the simulated population structure. Fig. S2. Effect of parameter *N*_win_ on prediction accuracy in the simulation. Fig. S3. Effect of smoothing parameter spar on ***w***-vector curves and LD block breakpoint identification. Fig. S4. Partitioning of reference (training) and validation populations in cross-validation. Fig. S5. Genetic correlations between Yorkshire (YY) and Landrace (LL) in analyzed traits. Fig. S6. Breakpoints of regional partitioning strategies employed by the mbBayesABLD (red) and mbBayesAB-lava (blue) models on a chromosome. Fig. S7. Individuals clustered based on principal components analysis using genotypes. Fig. S8. Changes in correlations of linkage disequilibrium coefficient (r) between subgroups on distance between two single nucleotide polymorphism (SNP) markers. Table S1. Prediction accuracy and unbiasedness under different iteration times in the mbBayesABLD model. Table S2. Breeds included in reference populations for multiple breed genomic prediction. Table S3. Genetic correlations between Yorkshire and Landrace in a multi-trait model. Table S4. Genetic correlations between bean panels in a multi-trait model. Table S5. Computational speed (min) of multibreed genomic prediction models in the simulation study. Table S6. Peak memory usage (MB) of multibreed genomic prediction models in the simulation study. Table S7. The accuracies of different models in the simulated datasets. Table S8. The unbiasedness of different models in the simulated datasets. Table S9. The accuracies of different models in real datasets. Table S10. The unbiasedness of different models in real datasets. Table S11. Allele frequency correlations (upper triangle) and persistence of the LD phase (lower triangle) between breeds in simulated and real datasets. Supplemental Methods

## Data Availability

The source code of mbBayesABLD software, together with all code and programs required to reproduce the results of this study, is publicly available in a unified GitHub repository: https://github.com/CAU-TeamLiuJF/MBGP_CV.

## Abbreviations

100SdW: 100-Seed weight
ADP: Andean diversity panel
CV: Cross-validation
LD: Linkage disequilibrium
LL: Landrace
MBGP: Multiple breeds for genomic prediction
MS: Marbling score
PCA: Principal component analysis
PFAI: The proportion of fat areas in the image
VEC: The newly composed climbing bean panel
VEF: The elite Andean breeding panel
YY: Yorkshire
