# Supplementary material for: Improving multibreed genomic prediction for breeds with small populations by modeling heterogeneous genetic (co)variance blockwise accounting for linkage disequilibrium
Source: J Anim Sci Biotechnol. 2025 Dec 20;16:176. doi: 10.1186/s40104-025-01303-9 (PMC12717733; doi:10.1186/s40104-025-01303-9)
Supplement: Supplementary file 1 — Additional file 1: Fig. S1. Schematic representation of the simulated population structure. Fig. S2. Effect of parameter Nwin on prediction accuracy in the simulation. Fig. S3. Effect of smoothing parameter spar on w-vector curves and LD block breakpoint identification. Fig. S4. Partitioning of reference (training) and validation populations in cross-validation. Fig. S5. Genetic correlations between Yorkshire (YY) and Landrace (LL) in analyzed traits. Fig. S6. Breakpoints of regional partitioning strategies employed by the mbBayesABLD (red) and mbBayesAB-lava (blue) models on a chromosome. Fig. S7. Individuals clustered based on principal components analysis using genotypes. Fig. S8. Changes in correlations of linkage disequilibrium coefficient (r) between subgroups on distance between two single nucleotide polymorphism (SNP) markers. Table S1. Prediction accuracy and unbiasedness under different iteration times in the mbBayesABLD model. Table S2. Breeds included in reference populations for multiple breed genomic prediction. Table S3. Genetic correlations between Yorkshire and Landrace in a multi-trait model. Table S4. Genetic correlations between bean panels in a multi-trait model. Table S5. Computational speed (min) of multibreed genomic prediction models in the simulation study. Table S6. Peak memory usage (MB) of multibreed genomic prediction models in the simulation study. Table S7. The accuracies of different models in the simulated datasets. Table S8. The unbiasedness of different models in the simulated datasets. Table S9. The accuracies of different models in real datasets. Table S10. The unbiasedness of different models in real datasets. Table S11. Allele frequency correlations (upper triangle) and persistence of the LD phase (lower triangle) between breeds in simulated and real datasets. Supplemental Methods [file 40104_2025_1303_MOESM1_ESM.docx]

# Additional file 1

## Supplemental Figures

**Fig.** **S1.** Schematic representation of the simulated population structure

**Fig. S2.** Effect of parameter *N*_win_ on prediction accuracy in the simulation

**Fig. S3.** Effect of smoothing parameter spar on ***w***-vector curves and LD block breakpoint identification

**Fig. S4.** Partitioning of reference (training) and validation populations in cross-validation

**Fig. S5.** Genetic correlations between Yorkshire (YY) and Landrace (LL) in analyzed traits

**Fig. S6.** Breakpoints of regional partitioning strategies employed by the mbBayesABLD (red) and mbBayesAB-lava (blue) models on a chromosome

**Fig. S7.** Individuals clustered based on principal components analysis using genotypes

**Fig. S8.** Changes in correlations of linkage disequilibrium coefficient (r) between subgroups on distance between two single nucleotide polymorphism (SNP) markers

## Supplemental Tables

**Table S1.** Prediction accuracy and unbiasedness using different iteration times in the mbBayesABLD model

**Table S2.** Breeds included in reference populations for multiple breed genomic prediction

**Table S3**. Genetic correlations between Yorkshire and Landrace in multi-trait joint prediction models

**Table S4.** Genetic correlations between bean panels in a multi-trait model

**Table S5.** Computational speed (min) of multibreed genomic prediction models in the simulation study

**Table S6.** Peak memory usage (MB) of multibreed genomic prediction models in the simulation study

**Table S7.** The accuracies of different models in the simulated datasets

**Table S8.** The unbiasedness of different models in the simulated datasets

**Table S9.** The accuracies of different models in real datasets

**Table S10.** The unbiasedness of different models in real datasets

**Table S11.** Allele frequency correlations (upper triangle) and persistence of the LD phase (lower triangle) between breeds in simulated and real datasets

## Supplemental Methods

Cross-validation and prediction accuracy

Genotype and phenotype simulations

Real data for model validation

Derivation of posterior distribution

GBLUP models for accuracy comparison

**References**

# Supplemental Figures


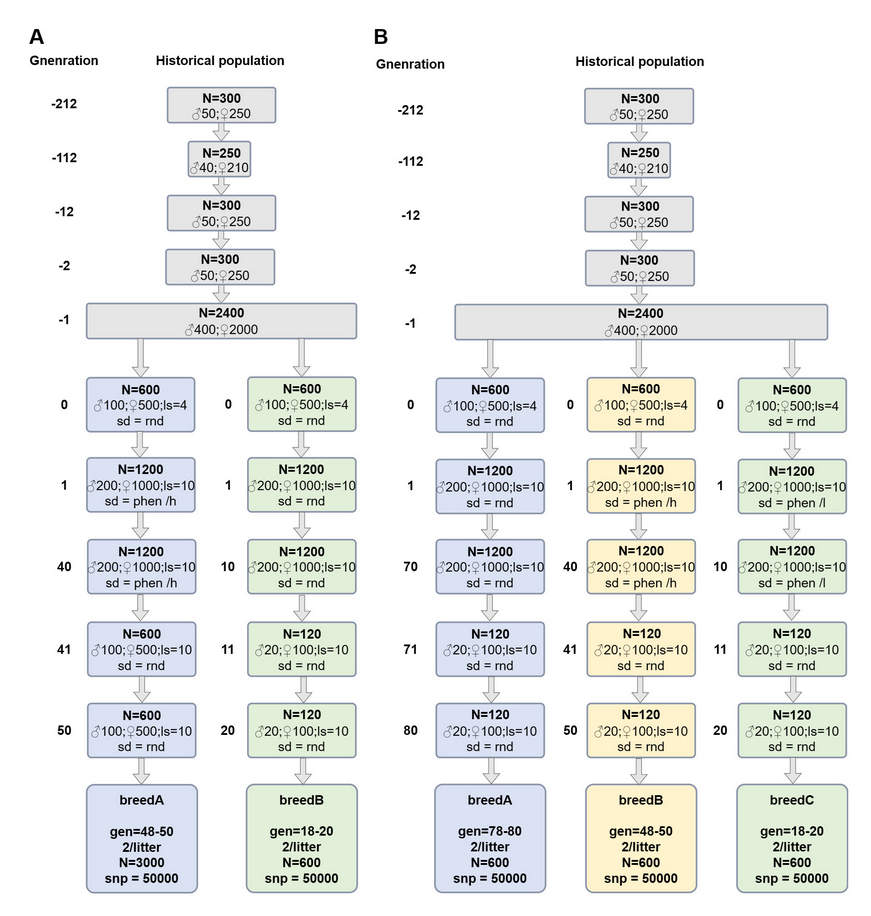


Fig. S1 Schematic representation of the simulated population structure. A Simulate a larger-sized breed A and a smaller-sized breed B, resembling the joint prediction of a commercial breed and a local breed. B Simulate three breeds with small population sizes, resembling the joint prediction of multiple local breeds





**Fig. S2** Effect of parameter *N*_win_ on prediction accuracy in the simulation. *N*_win_ denotes the number of SNPs furthest from marker *k* that are considered when calculating the correlation coefficient


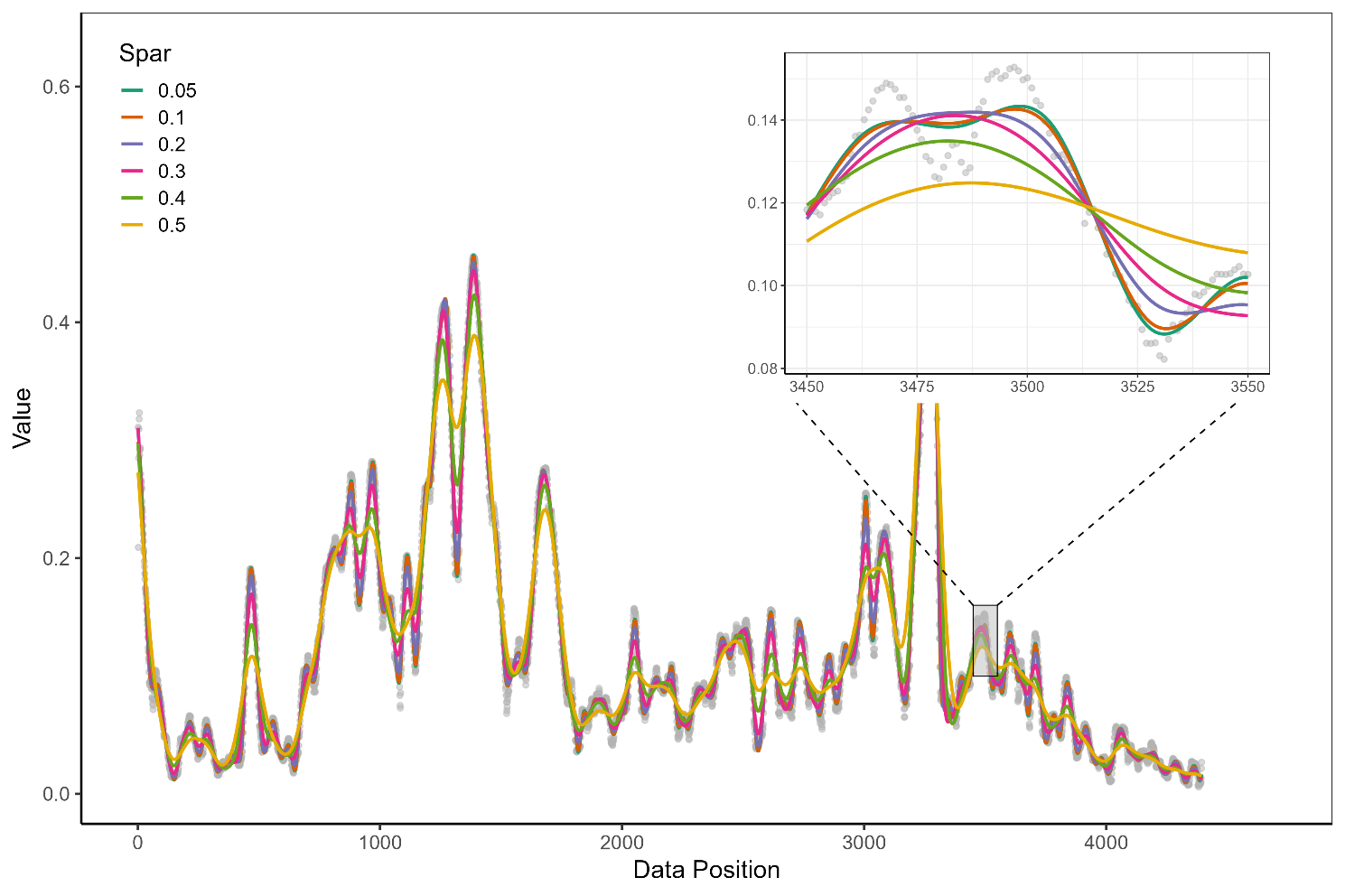


**Fig. S3** Effect of smoothing parameter spar on *w*-vector curves and LD block breakpoint identification. The ***w***-vector represents the metric for linkage between subblocks along a chromosome, with theoretical values ranging from 0 to 1. Curves were generated using the smooth.spline function from R’s *stats* package. Chromosome 1 for real pig genotype data were used for LD calculation and visualization. A spar value of 0.2 provided moderate smoothing and enabled clearer identification of LD block breakpoints


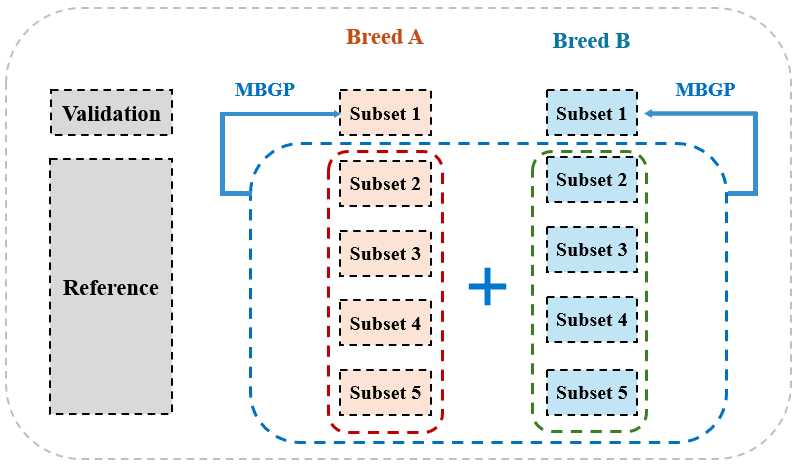


**Fig. S4** Partitioning of reference (training) and validation populations in cross-validation. The estimates of local genetic correlations were obtained for the marbling score (MS) and proportion of fat areas in the image (PFAI) traits when applying the mbBayesABLD model for joint prediction



**Fig. S5** Genetic correlations between Yorkshire (YY) and Landrace (LL) in analyzed traits. The estimates of local genetic correlations were obtained for the marbling score (MS) and proportion of fat areas in the image (PFAI) traits when applying the mbBayesABLD model for joint prediction



Fig. S6 Breakpoints of regional partitioning strategies employed by the mbBayesABLD (red) and mbBayesAB-lava (blue) models on a chromosome. The reference panel consists of Chromosome 2 from the combined population of Yorkshire (YY) and Landrace (LL). The gray scatter represents the linkage disequilibrium measure between two sub-blocks, assuming the SNP is the partitioning breakpoint. The black solid line shows the smoothed measure after applying a cubic spline function





Fig. S7 Individuals clustered based on principal components analysis using genotypes. A Simulated dataset of two breeds including A and B. B Simulated dataset of three breeds including A, B and C. C Real pig dataset including Yorkshire (YY) and Landrace (LL). D Real bean dataset including newly composed climbing bean panel (VEC), Andean diversity panel (ADP) and elite Andean breeding panel (VEF)





**Fig. S8** Changes in correlations of linkage disequilibrium coefficient (*r*) between subgroups on distance between two single nucleotide polymorphism (SNP) markers. **A** Simulated dataset for a scenario involving two breeds. **B** Simulated dataset for a scenario involving three breeds. **C** Real pig dataset. **D** Real bean dataset.

# Supplemental Tables

Table S1 Prediction accuracy and unbiasedness using different iteration times in the mbBayesABLD model

| **Dataset** | **Reference** | **Trait** | **accuracy** | | **bias** | |
| --- | --- | --- | --- | --- | --- | --- |
|  |  |  | **30000** | **110000** | **30000** | **110000** |
| Pig | YY + LL | PFAI | 0.190±0.066 | 0.185±0.068 | 0.678±0.322 | 0.664±0.317 |
| Bean | VEC + VEF + ADP | Yield | 0.282±0.117 | 0.280±0.114 | 0.507±0.353 | 0.503±0.351 |

YY, Yorkshire; LL, Landrace; VEC, climbing bean panel; ADP, Andean diversity panel; VEF, elite Andean breeding panel. ‘YY + LL’ represents the reference population consisting of two breeds, YY and LL. We analyzed a trait in two real datasets using the mbBayesABLD model. The results showed that there was no significant difference (P<0.05) between prediction accuracy and unbiasedness when the number of iterations of the Bayesian model increased from 30000 to 110000

**Table S2** Breeds included in reference populations for multiple breed genomic prediction

| **Type** | **Dataset** | **Breeds in reference population** |
| --- | --- | --- |
| Simulation | Two breeds | A + B |
|  | Three breeds | A + B, A + C, A + B + C |
| Real | Pig | YY + LL |
|  | Bean | VEC + ADP, VEC + VEF, VEC + ADP + VEF |

YY, Yorkshire; LL, Landrace; VEC, climbing bean panel; ADP, Andean diversity panel; VEF, elite Andean breeding panel. ‘A + B’ represents the reference population consisting of two breeds, breed A and breed B

Table S3 Genetic correlations between Yorkshire and Landrace in multi-trait joint prediction models

| **Model^1^** | **MS** | **PFAI** |
| --- | --- | --- |
| MTGBLUP | -0.74 ± 0.35 | -0.76 ± 0.22 |
| mbBayesAB-fix | -0.02 ± 0.03 | -0.03 ± 0.03 |
| mbBayesAB-lava | -0.03 ± 0.04 | -0.04 ± 0.03 |
| mbBayesABLD | -0.01 ± 0.02 | -0.03 ± 0.03 |

^1^All models treat the same trait from different breeds (Yorkshire and Landrace) as genetically correlated but distinct traits. In the Bayesian models, we divide the genome into non-overlapping blocks. The genetic correlation shown here is the global genetic correlation between breeds

MS, marbling score; PFAI, proportion of fat areas in the image

Table S4 Genetic correlations between bean panels in multi-trait joint prediction models

| **Trait**^1^ | **Breed**^2^ | **MTGBLUP**^3^ | **mbBayesAB-fix** | **mbBayesAB-lava** | **mbBayesABLD** |
| --- | --- | --- | --- | --- | --- |
| 100SdW | ADP-VEF | 0.620±0.155 | 0.548±0.062 | 0.561±0.061 | 0.583±0.055 |
| 100SdW | VEC-ADP | 0.459±0.12 | 0.608±0.035 | 0.590±0.036 | 0.575±0.039 |
| 100SdW | VEC-VEF | 0.500±0.118 | 0.444±0.083 | 0.413±0.081 | 0.431±0.076 |
| Yield | ADP-VEF | 0.465±0.395 | 0.069±0.086 | 0.061±0.093 | 0.070±0.099 |
| Yield | VEC-ADP | -0.253±0.732 | -0.108±0.046 | -0.108±0.045 | -0.060±0.053 |
| Yield | VEC-VEF | 0.371±0.333 | 0.073±0.057 | 0.076±0.055 | 0.093±0.057 |

^1^100SdW, 100-seed weight; Yield, bean yield per hectare

^2^Genetic correlation were calculated using the three-trait form of the mbBayesABLD model. The reference population consisting of three panels: VEC, VEF and ADP. ADP-VEF indicates that the genetic correlation in this row represents the estimated genetic correlation between ADP and VEF

^3^All models treat the same trait from different panels as genetically correlated but distinct traits. In the Bayesian models, we divide the genome into non-overlapping blocks. The genetic correlation shown here is the global genetic correlation between breeds

**Table S5** Computational speed (min) of multibreed genomic prediction models in the simulation study

| **Ref**^1^ | **N_ref_**^2^ | **Dist**^3^ | **Rg**^4^ | **STGBLUP** | **BayesR** | **MTGBLUP** | **mbBayesAB-fix** | **mbBayesAB-lava** | **mbBayesABLD** |
| --- | --- | --- | --- | --- | --- | --- | --- | --- | --- |
| A-B | 2880 | identical | 0.2 | 17.09±2.35 | 40.35±6.09 | 101.18±12.36 | 98.88±5.65 | 98.63±5.46 | 99.68±6.50 |
|  |  |  | 0.5 | 17.04±2.20 | 39.57±6.19 | 101.69±13.24 | 92.99±20.82 | 98.98±7.10 | 98.17±7.04 |
|  |  |  | 0.8 | 16.62±1.98 | 39.47±6.16 | 100.98±11.49 | 100.43±7.91 | 100.33±7.12 | 100.74±7.93 |
|  |  | uniform | 0.2 | 17.13±2.03 | 38.99±6.34 | 100.34±16.68 | 96.86±5.90 | 97.39±6.22 | 97.73±6.10 |
|  |  |  | 0.5 | 17.12±1.89 | 38.45±6.52 | 99.10±15.94 | 98.22±7.16 | 98.72±7.55 | 99.08±8.45 |
|  |  |  | 0.8 | 16.87±2.15 | 38.99±6.48 | 98.57±13.32 | 100.08±6.8 | 98.42±5.52 | 102.03±6.66 |
| A-B | 960 | identical | 0.2 | 0.67±0.10 | 25.93±3.84 | 7.79±16.30 | 42.09±1.55 | 43.27±3.29 | 43.67±2.79 |
|  |  | uniform | 0.2 | 0.70±0.12 | 25.19±3.99 | 3.78±3.30 | 43.48±3.76 | 42.64±2.14 | 43.3±2.76 |
| A-C | 960 | identical | 0.2 | 0.70±0.13 | 24.84±3.95 | 18.09±30.94 | 42.75±2.3 | 41.99±1.54 | 42.71±2.28 |
|  |  | uniform | 0.2 | 0.67±0.11 | 25.98±3.57 | 14.43±27.98 | 42.65±2.32 | 42.41±1.94 | 43.85±3.04 |
| A-B-C | 1440 | identical | 0.2 | 2.17±0.31 | 32.83±0.89 | 336.56±360.11 | 74.66±3.81 | 74.46±3.44 | 71.84±10.30 |
|  |  | uniform | 0.2 | 2.08±0.37 | 32.3±3.04 | 321.39±371.05 | 71.86±10.2 | 66.84±14.09 | 71.09±12.58 |

Data are presented as mean±SD from 20 independent simulation replicates

^1^The breeds included in the reference populations. For example, YY-LL indicates that the reference was composed of Yorkshire (YY) and Landrace (LL)

^2^The number of individuals in reference populations

^3^Distribution of genetic correlations among marker effects in 10 genomic regions selected for simulated phenotypes. Identical, a constant genetic correlation across all regions; uniform, genetic correlations sampled from a uniform distribution U(-1, 1) within each interval

^4^Average genetic correlation in 10 genomic regions selected for simulated phenotypes

Note: In the MTGBLUP model, the variance component estimates in certain cases did not reach convergence criteria and hit the maximum number of iterations (200) set by the DMU software

**Table S6** Peak memory usage (MB) of multibreed genomic prediction models in the simulation study

| **Ref**^1^ | **N_ref_**^2^ | **Dist**^3^ | **Rg**^4^ | **STGBLUP** | **BayesR** | **MTGBLUP** | **mbBayesAB-fix** | **mbBayesAB-lava** | **mbBayesABLD** |
| --- | --- | --- | --- | --- | --- | --- | --- | --- | --- |
| A-B | 2880 | identical | 0.2 | 477.26±0.61 | 136.07±1.16 | 1645.20±0.64 | 2186.30±6.72 | 2190.87±10.98 | 2190.88±10.98 |
|  |  |  | 0.5 | 477.04±0.47 | 135.74±0.73 | 1489.57±439.36 | 2190.83±11.01 | 2190.82±11.00 | 2190.50±10.85 |
|  |  |  | 0.8 | 477.32±0.66 | 136.01±1.19 | 1430.52±535.43 | 2190.85±11.05 | 2190.84±11.01 | 2190.88±11.27 |
|  |  | uniform | 0.2 | 477.28±0.65 | 135.71±0.69 | 1645.32±0.70 | 2190.88±11.04 | 2190.82±11.23 | 2190.88±11.04 |
|  |  |  | 0.5 | 477.22±0.55 | 136.77±1.91 | 1612.78±231.29 | 2192.38±11.89 | 2190.85±11.12 | 2190.85±10.98 |
|  |  |  | 0.8 | 477.23±0.59 | 136.18±1.37 | 1645.71±0.66 | 2190.87±11.04 | 2190.85±11.02 | 2102.82±297.73 |
| A-B | 960 | identical | 0.2 | 63.62±0.70 | 67.10±0.00 | 179.45±0.00 | 752.19±6.63 | 750.67±6.44 | 752.03±6.58 |
|  |  | uniform | 0.2 | 63.36±0.50 | 67.38±0.62 | 180.06±0.62 | 752.20±6.62 | 752.17±6.62 | 753.76±6.06 |
| A-C | 960 | identical | 0.2 | 115.87±18.85 | 83.62±1.65 | 641.61±426.27 | 1125.97±9.88 | 1125.96±9.90 | 1125.95±9.91 |
|  |  | uniform | 0.2 | 120.86±0.00 | 83.02±0.92 | 905.23±125.25 | 1127.82±10.30 | 1124.18±10.65 | 1125.99±9.92 |
| A-B-C | 1440 | identical | 0.2 | 63.60±0.66 | 67.29±0.53 | 179.70±0.54 | 752.21±6.63 | 752.28±6.70 | 752.36±6.56 |
|  |  | uniform | 0.2 | 63.04±1.58 | 67.46±0.72 | 172.23±35.32 | 750.12±5.70 | 751.96±6.62 | 752.70±7.27 |

^1^The breeds included in the reference populations. For example, YY-LL indicates that the reference was composed of Yorkshire (YY) and Landrace (LL)

^2^The number of individuals in reference populations

^3^Distribution of genetic correlations among marker effects in 10 genomic regions selected for simulated phenotypes. Identical, a constant genetic correlation across all regions; uniform, genetic correlations sampled from a uniform distribution U(-1, 1) within each interval

^4^Average genetic correlation in 10 genomic regions selected for simulated phenotypes

Note: In the MTGBLUP model, the variance component estimates in certain cases did not reach convergence criteria and hit the maximum number of iterations (200) set by the DMU software

| **Ref**^1^ | **Val**^2^ | **Dist**^3^ | **Rg**^4^ | **BayesR** | **STGBLUP** | **MTGBLUP** | **mbBayesAB-fix** | **mbBayesAB-lava** | **mbBayesABLD** |
| --- | --- | --- | --- | --- | --- | --- | --- | --- | --- |
| A-B | A | identical | 0.2 | 0.641±0.006^ab^ | 0.605±0.006^ab^ | 0.625±0.006^ab^ | 0.654±0.006^ab^ | 0.654±0.006^ab^ | 0.657±0.006^a^ |
|  |  |  | 0.5 | 0.643±0.006^ab^ | 0.607±0.006^ab^ | 0.625±0.006^ab^ | 0.654±0.006^ab^ | 0.654±0.006^ab^ | 0.657±0.006^a^ |
|  |  |  | 0.8 | 0.645±0.006^ab^ | 0.608±0.006^ab^ | 0.624±0.006^b^ | 0.653±0.006^ab^ | 0.653±0.006^ab^ | 0.656±0.006^a^ |
|  |  | uniform | 0.2 | 0.641±0.006^ab^ | 0.612±0.006^ab^ | 0.633±0.006^ab^ | 0.656±0.006^ab^ | 0.656±0.006^ab^ | 0.659±0.006^a^ |
|  |  |  | 0.5 | 0.641±0.007^ab^ | 0.612±0.007^ab^ | 0.630±0.007^b^ | 0.652±0.007^ab^ | 0.652±0.007^ab^ | 0.655±0.007^a^ |
|  |  |  | 0.8 | 0.640±0.007^ab^ | 0.610±0.006^ab^ | 0.627±0.006^b^ | 0.649±0.007^ab^ | 0.649±0.007^ab^ | 0.653±0.007^ab^ |
|  | B | identical | 0.2 | 0.400±0.010^ab^ | 0.401±0.012^ab^ | 0.447±0.012^a^ | 0.451±0.013^b^ | 0.450±0.013^b^ | 0.454±0.014^b^ |
|  |  |  | 0.5 | 0.416±0.010^ab^ | 0.414±0.012^ab^ | 0.449±0.012^b^ | 0.452±0.013^b^ | 0.451±0.013^b^ | 0.455±0.014^ab^ |
|  |  |  | 0.8 | 0.432±0.010^ab^ | 0.426±0.012^ab^ | 0.452±0.013^b^ | 0.454±0.014^ab^ | 0.452±0.014^b^ | 0.457±0.014^ab^ |
|  |  | uniform | 0.2 | 0.383±0.017^ab^ | 0.378±0.018^ab^ | 0.425±0.020^ab^ | 0.439±0.019^ab^ | 0.440±0.019^ab^ | 0.441±0.018^ab^ |
|  |  |  | 0.5 | 0.398±0.016^ab^ | 0.394±0.016^ab^ | 0.426±0.019^ab^ | 0.437±0.019^ab^ | 0.439±0.018^ab^ | 0.439±0.019^ab^ |
|  |  |  | 0.8 | 0.407±0.016^ab^ | 0.401±0.016^ab^ | 0.429±0.018^a^ | 0.439±0.018^ab^ | 0.440±0.018^ab^ | 0.441±0.018^ab^ |
| A-B | A | identical | 0.2 | 0.413±0.019^ab^ | 0.404±0.019^ab^ | 0.418±0.018^ab^ | 0.425±0.019^b^ | 0.427±0.020 | 0.428±0.020 |
|  |  | uniform | 0.2 | 0.400±0.013^ab^ | 0.396±0.012^ab^ | 0.402±0.014^ab^ | 0.410±0.014 | 0.409±0.015^b^ | 0.411±0.015 |
| A-C |  | identical | 0.2 | 0.407±0.018^ab^ | 0.402±0.016^ab^ | 0.419±0.017^ab^ | 0.424±0.019 | 0.425±0.019 | 0.426±0.019^b^ |
|  |  | uniform | 0.2 | 0.389±0.017^ab^ | 0.384±0.016^ab^ | 0.403±0.014^ab^ | 0.408±0.014 | 0.408±0.014^b^ | 0.411±0.016^b^ |
| A-B-C |  | identical | 0.2 | 0.392±0.018^ab^ | 0.386±0.018^ab^ | 0.416±0.017^ab^ | 0.427±0.019 | 0.429±0.019^ab^ | 0.430±0.020^ab^ |
|  |  | uniform | 0.2 | 0.377±0.014^ab^ | 0.374±0.013^ab^ | 0.396±0.016^ab^ | 0.411±0.014 | 0.409±0.014^b^ | 0.414±0.015^ab^ |

**Table S7** The accuracies of different models in the simulated datasets

^1^The breeds included in the reference populations. For example, YY-LL indicate that the reference was composed of Yorkshire (YY) and Landrace (LL)

^2^The source breeds of individuals in the results calculations

^3^Distribution of genetic correlations among marker effects in 10 genomic regions selected for simulated phenotypes. Identical, a constant genetic correlation across all regions; uniform, genetic correlations sampled from a uniform distribution U(-1, 1) within each interval

^4^Average genetic correlation in 10 genomic regions selected for simulated phenotypes

The superscripts ‘a’ indicates significant differences in mean compared to w-STGBLUP, while ‘b’ indicates significant differences in mean compared to w-BayesABLD (*P* < 0.05)

**Table S8** The unbiasedness of different models in the simulated datasets

| **Ref**^1^ | **Val**^2^ | **Dist**^3^ | **Rg**^4^ | **BayesR** | **STGBLUP** | **MTGBLUP** | **mbBayesAB-fix** | **mbBayesAB-lava** | **mbBayesABLD** |
| --- | --- | --- | --- | --- | --- | --- | --- | --- | --- |
| A-B | A | identical | 0.2 | 0.048±0.016^ab^ | 0.053±0.018^ab^ | 0.010±0.016^ab^ | -0.057±0.012^ab^ | -0.054±0.013^ab^ | -0.059±0.012^ab^ |
|  |  |  | 0.5 | 0.046±0.016^ab^ | 0.052±0.017^ab^ | 0.010±0.016^ab^ | -0.057±0.012^ab^ | -0.055±0.013^ab^ | -0.060±0.013^ab^ |
|  |  |  | 0.8 | 0.044±0.016^ab^ | 0.050±0.017^ab^ | 0.011±0.016^ab^ | -0.058±0.013^ab^ | -0.055±0.013^ab^ | -0.061±0.013^ab^ |
|  |  | uniform | 0.2 | 0.042±0.016^ab^ | 0.049±0.016^ab^ | -0.004±0.014^ab^ | -0.061±0.012^ab^ | -0.057±0.012^ab^ | -0.065±0.012^ab^ |
|  |  |  | 0.5 | 0.034±0.016^ab^ | 0.041±0.017^ab^ | -0.007±0.014^ab^ | -0.066±0.012^ab^ | -0.061±0.012^ab^ | -0.069±0.012^ab^ |
|  |  |  | 0.8 | 0.031±0.016^ab^ | 0.040±0.016^ab^ | -0.007±0.014^ab^ | -0.066±0.012^ab^ | -0.062±0.012^ab^ | -0.070±0.012^ab^ |
|  | B | identical | 0.2 | -0.336±0.020^ab^ | -0.292±0.023^a^ | 0.103±0.065^ab^ | -0.288±0.025^ab^ | -0.285±0.026^ab^ | -0.289±0.026^ab^ |
|  |  |  | 0.5 | -0.316±0.021^ab^ | -0.276±0.024^ab^ | 0.105±0.065^ab^ | -0.287±0.025^ab^ | -0.284±0.027^ab^ | -0.287±0.026^ab^ |
|  |  |  | 0.8 | -0.298±0.021^a^ | -0.261±0.024^ab^ | 0.106±0.064^ab^ | -0.287±0.026^ab^ | -0.283±0.027^ab^ | -0.286±0.026^ab^ |
|  |  | uniform | 0.2 | -0.380±0.038^ab^ | -0.343±0.038^ab^ | 0.123±0.135^ab^ | -0.310±0.037^ab^ | -0.304±0.037^ab^ | -0.310±0.036^ab^ |
|  |  |  | 0.5 | -0.359±0.036^ab^ | -0.322±0.036^a^ | 0.102±0.096^ab^ | -0.308±0.038^ab^ | -0.302±0.036^ab^ | -0.309±0.037^ab^ |
|  |  |  | 0.8 | -0.350±0.033^ab^ | -0.312±0.035^a^ | 0.082±0.082^ab^ | -0.307±0.036^ab^ | -0.301±0.035^ab^ | -0.309±0.035^ab^ |
| A-B | A | identical | 0.2 | 0.200±0.091^ab^ | -0.040±0.051^ab^ | 0.012±0.075^ab^ | -0.317±0.030^a^ | -0.311±0.033^ab^ | -0.315±0.031^ab^ |
|  |  | uniform | 0.2 | 0.364±0.122^ab^ | 0.018±0.053^ab^ | 0.108±0.070^ab^ | -0.330±0.024^ab^ | -0.325±0.025^ab^ | -0.329±0.026^ab^ |
| A-C |  | identical | 0.2 | 0.383±0.109^ab^ | 0.019±0.051^ab^ | 0.011±0.060^ab^ | -0.316±0.030^ab^ | -0.311±0.031^ab^ | -0.314±0.030^ab^ |
|  |  | uniform | 0.2 | 0.608±0.181^ab^ | 0.062±0.054^ab^ | 0.133±0.087^b^ | -0.329±0.024^ab^ | -0.325±0.023^ab^ | -0.331±0.024^ab^ |
| A-B-C |  | identical | 0.2 | 0.008±0.053^ab^ | -0.052±0.045^ab^ | -0.010±0.088^ab^ | -0.313±0.032^ab^ | -0.305±0.031^ab^ | -0.309±0.032^ab^ |
|  |  | uniform | 0.2 | 0.123±0.067^ab^ | 0.002±0.046^ab^ | 0.082±0.096^ab^ | -0.325±0.023^ab^ | -0.322±0.023^ab^ | -0.324±0.024^ab^ |

^1^The breeds included in the reference populations. For example, YY-LL indicate that the reference was composed of Yorkshire (YY) and Landrace (LL).

^2^The source breeds of individuals in the results calculations

^3^Distribution of genetic correlations among marker effects in 10 genomic regions selected for simulated phenotypes. Identical, a constant genetic correlation across all regions; uniform, genetic correlations sampled from a uniform distribution U(-1, 1) within each interval

^4^Average genetic correlation in 10 genomic regions selected for simulated phenotypes

The superscripts ‘a’ indicates significant differences in mean compared to w-STGBLUP, while ‘b’ indicates significant differences in mean compared to w-BayesABLD (*P* < 0.05). Unbiasedness was represented by subtracting 1 from the regression coefficient. The values greater than 0 indicate a biased deflation prediction of EBVs, and smaller than 0 indicate a biased inflation prediction of EBVs

**Table S9** The accuracies of different models in real datasets

| **Ref**^1^ | **Val**^2^ | **Trait**^3^ | **BayesR** | **STGBLUP** | **MTGBLUP** | **mbBayesAB-fix** | **mbBayesAB-lava** | **mbBayesABLD** |
| --- | --- | --- | --- | --- | --- | --- | --- | --- |
| YY-LL | YY | MS | 0.157±0.081^ab^ | 0.155±0.080a^b^ | 0.170±0.080^b^ | 0.180±0.082a^b^ | 0.170±0.080^b^ | 0.209±0.086a |
|  |  | PFAI | 0.130±0.074a^b^ | 0.130±0.073a^b^ | 0.158±0.070^b^ | 0.161±0.075^b^ | 0.160±0.075^b^ | 0.189±0.076a |
|  | LL | MS | 0.195±0.113 | 0.128±0.107a^b^ | 0.188±0.119 | 0.203±0.113 | 0.201±0.114 | 0.223±0.110a^b^ |
|  |  | PFAI | 0.155±0.118a^b^ | 0.128±0.117a^b^ | 0.196±0.131 | 0.198±0.116 | 0.197±0.116 | 0.220±0.119a^b^ |
| VEC-ADP | VEC | Yield | 0.214±0.095^b^ | 0.247±0.100a^b^ | 0.144±0.155a^b^ | 0.273±0.106a^b^ | 0.267±0.113a^b^ | 0.285±0.099a^b^ |
|  |  | 100SdW | 0.658±0.073a^b^ | 0.659±0.072a^b^ | 0.681±0.068^b^ | 0.690±0.065a | 0.689±0.067a | 0.685±0.065^b^ |
| VEC-VEF |  | Yield | 0.280±0.104a^b^ | 0.281±0.109a^b^ | 0.116±0.128a^b^ | 0.282±0.105a^b^ | 0.274±0.107a^b^ | 0.310±0.097a^b^ |
|  |  | 100SdW | 0.667±0.069a^b^ | 0.679±0.070^b^ | 0.689±0.067a | 0.689±0.068a | 0.688±0.068a | 0.694±0.066a^b^ |
| VEC-ADP-VEF |  | Yield | 0.208±0.103^b^ | 0.279±0.105a | 0.074±0.149a^b^ | 0.275±0.109a^b^ | 0.273±0.112a^b^ | 0.281±0.102a^b^ |
|  |  | 100SdW | 0.650±0.069a^b^ | 0.651±0.073a^b^ | 0.687±0.067a | 0.691±0.063a | 0.693±0.063a | 0.685±0.064 |

^1^The breeds included in the reference populations. For example, YY-LL indicate that the reference was composed of Yorkshire (YY) and Landrace (LL)

^2^The source breeds of individuals in the results calculations

^3^MS, marbling score; PFAI, proportion of fat areas in the image; 100SdW, 100-seed weight; Yield, yield

The superscripts a indicates significant differences in mean compared to w-STGBLUP, while b indicates significant differences in mean compared to w-BayesABLD (*P* < 0.05). Unbiasedness was represented by subtracting 1 from the regression coefficient. The values greater than 0 indicate a biased deflation prediction of EBVs, and smaller than 0 indicate a biased inflation prediction of EBVs

**Table S10** The unbiasedness of different models in real datasets

| **Ref**^1^ | **Val**^2^ | **Trait**^3^ | **BayesR** | **STGBLUP** | **MTGBLUP** | **mbBayesAB-fix** | **mbBayesAB-lava** | **mbBayesABLD** |
| --- | --- | --- | --- | --- | --- | --- | --- | --- |
| YY-LL | YY | MS | 0.812±1.294^ab^ | 0.101±0.807^ab^ | 0.141±0.764^b^ | -0.324±0.300^ab^ | -0.336±0.306^ab^ | -0.261±0.317^ab^ |
|  |  | PFAI | 0.905±1.415^ab^ | 0.123±0.842^ab^ | 0.205±0.865^b^ | -0.389±0.323^a^b | -0.383±0.325^ab^ | -0.318±0.337^ab^ |
|  | LL | MS | 1.111±1.696^ab^ | 0.275±1.264^b^ | 0.402±1.300^b^ | -0.128±0.525^a^ | -0.179±0.511^ab^ | -0.057±0.519^ab^ |
|  |  | PFAI | 1.095±2.353^b^ | 0.519±1.633^b^ | 0.615±2.411^b^ | -0.093±0.597^a^ | -0.134±0.570^ab^ | -0.032±0.609^ab^ |
| VEC-ADP | VEC | Yield | -0.406±0.488^a^ | -0.282±0.446^ab^ | -1.108±0.858^ab^ | -0.449±0.303^ab^ | -0.460±0.318^ab^ | -0.466±0.290^ab^ |
|  |  | 100SdW | -0.039±0.109^ab^ | -0.044±0.117^a^ | -0.009±0.112^ab^ | -0.100±0.109^ab^ | -0.095±0.110^ab^ | -0.108±0.111^ab^ |
| VEC-VEF |  | Yield | -0.619±0.222^ab^ | -0.488±0.261^ab^ | -0.994±0.012^ab^ | -0.316±0.365^a^ | -0.325±0.377^a^ | -0.313±0.341^ab^ |
|  |  | 100SdW | -0.032±0.107^ab^ | -0.031±0.102^ab^ | -0.005±0.109^b^ | -0.053±0.112^a^ | -0.046±0.114^ab^ | -0.053±0.111^a^ |
| VEC-ADP-VEF |  | Yield | -0.645±0.292^ab^ | -0.456±0.276^ab^ | -0.995±0.039^ab^ | -0.451±0.30^3ab^ | -0.445±0.317^ab^ | -0.478±0.293^ab^ |
|  |  | 100SdW | -0.042±0.107^a^ | -0.044±0.106^a^ | -0.010±0.109^b^ | -0.103±0.107^ab^ | -0.095±0.109^ab^ | -0.109±0.108^ab^ |

^1^The breeds included in the reference populations. For example, YY-LL indicate that the reference was composed of Yorkshire (YY) and Landrace (LL)

^2^The source breeds of individuals in the results calculations

^3^MS, marbling score; PFAI, proportion of fat areas in the image; 100SdW, 100-seed weight; Yield, yield

The superscripts a indicates significant differences in mean compared to w-STGBLUP, while b indicates significant differences in mean compared to w-BayesABLD (*P* < 0.05). Unbiasedness was represented by subtracting 1 from the regression coefficient. The values greater than 0 indicate a biased deflation prediction of EBVs, and smaller than 0 indicate a biased inflation prediction of EBVs

Table S11 Allele frequency correlations (upper triangle) and persistence of the LD phase (lower triangle) between breeds in simulated and real datasets

| **Breed** | **Two breeds** | | **Three breeds** | | | **Pig** | | **Bean** | | |
| --- | --- | --- | --- | --- | --- | --- | --- | --- | --- | --- |
|  | **A** | **B** | **A** | **B** | **C** | **YY** | **LL** | **VEC** | **VEF** | **ADP** |
| A | - | 0.21 ± 0.06^1^ |  |  |  |  |  |  |  |  |
| B | 0.62 ± 0.02 | - |  |  |  |  |  |  |  |  |
| A |  |  | - | 0.21 ± 0.02 | 0.40 ± 0.02 |  |  |  |  |  |
| B |  |  | 0.57 ± 0.01 | - | 0.14 ± 0.02 |  |  |  |  |  |
| C |  |  | 0.63 ± 0.01 | 0.55 ± 0.01 | - |  |  |  |  |  |
| YY |  |  |  |  |  | - | 0.44 |  |  |  |
| LL |  |  |  |  |  | 0.67 | - |  |  |  |
| VEC |  |  |  |  |  |  |  | - | 0.02 | -0.04 |
| VEF |  |  |  |  |  |  |  | 0.69 | - | -0.02 |
| ADP |  |  |  |  |  |  |  | 0.63 | 0.74 | - |

^1^The simulation was repeated 20 times, and the results are presented as mean ± standard deviation

YY, Yorkshire; LL, Landrace; VEC, newly composed climbing bean panel; ADP, Andean diversity panel; VEF, elite Andean breeding panel

The persistence of the LD phase was calculated according to all pairs of SNPs within 10 Mbp

# Supplemental Methods

## Cross-validation and prediction accuracy

A five-fold cross-validation approach was employed to obtain accuracy and unbiasedness. The population, comprising individuals with both genotypic and phenotypic information, was randomly divided into five equal-sized subsets. One subset was designated as the validation set, with the phenotypic values of individuals set as missing, while the remaining individuals constituted the training set used for predicting the GEBVs of the individuals in validation. This process was repeated five times. The phenotypic values were adjusted using GBLUP model based on the complete dataset. It should be noted that in the dataset of the bean dataset, the response variable used was BLUEs. Hence, in this study, BLUEs were directly used as the corrected phenotypes (**y**_c_). Prediction accuracy was assessed by calculating the correlation coefficient between the GEBVs and **y**_c_, while unbiasedness was evaluated by the regression coefficient of the **y**_c_ on GEBVs.

In the case of the simulated and real pig datasets of two breeds, the reference population was formed by combining individuals from the two breeds. In the simulated three breed dataset, the scenario simulates the use of information from other breeds to improve the prediction accuracy of breed A. In addition to combining all three breeds in the reference population, we also investigate the impact of adding information from B and C separately in the reference population on the prediction accuracy of A. In the real bean dataset, our study aims at improving the prediction accuracy of climbing bean panel (VEC) by integrating data from bush type bean panels (ADP and VEF), which consistent previously established approaches [1]. But we only consider scenarios with a combined reference population of two or three breeds that including VEC. Additionally, to explore whether combining all panels can further improve the prediction accuracy of VEC, we also consider the combination of all five panels.

## Genotype and phenotype simulations

The strategy for genotype simulation of multiple breeds follows a previously established approach designed for multibreed scenarios [2]. Two (or three) different breeds were simulated using QMSim software [3] from a historical population that underwent 211 generations of random mating (Supplemental Figure S5). The historical population started with 300 individuals and gradually decreased to 250 in the 100 generation to create LD. In the 200 generation, the population size was increased gradually to 300, followed by 10 generations of random mating to consolidate LD. In the last generation of the historical population, the population size increased to 2400 individuals. In the entire historical population, there was no generation overlap, and random mating and random selection were practiced among individuals. The ratio of males to females was maintained at 1:5.

Two (or three) equally sized populations were randomly selected in the final generation of the historical population, with each consisting of 600 individuals. After one generation of random mating, the population size of both breeds increased to 1200 individuals, and the number of litter size was set to four. Then, different generations and individual selection criteria were applied to obtain different breeds with distinct gene frequencies (Supplemental Table S8). When simulating two breeds, breed A underwent 40 generations of random mating, while breed B underwent 10 generations. To induce variations in gene frequencies between two breeds, independent selection criteria were applied to breed A based on high phenotypic values, while breed B was selected based on low phenotypic values. In the case of simulating two breeds, we randomly selected two individuals from each litter in the last three generations of breeds A and B, resulting in a total number of 3000 and 600 individuals respectively for following analysis. When simulating three breeds, A, B and C underwent 70, 40 and 10 generations of random mating, respectively, and the individual selection criteria were random, high phenotypic value and low phenotypic value, respectively. In the simulation involving three breeds with small population, 600 individuals were randomly selected for each breed for phenotype simulation and genomic prediction.

Next, each breed underwent an additional 10 generations of random mating to consolidate the LD within each breed. In the two stages mentioned above, the number of offspring was set to 10. As in the historical population, these generations were discrete, with random mating and a ratio of males to females of 1:5.

There were 18 chromosomes for the simulated populations, each with a length of 100 cM, and 200,000 SNPs were randomly distributed on each chromosome (Supplemental Table S9). We included 100 randomly distributed QTL on each chromosome. The QTL effects were sampled from a random normal distribution. Among the segregating SNPs in both breeds, we partitioned each chromosome into segments of 10 cM based on their physical positions. Approximately 278 SNPs on average were randomly selected within each block, resulting in a total of 50,058 SNPs were obtained for phenotype simulation and genomic prediction analysis.

The phenotypes used in the analysis were simulated using self-developed R scripts. A total number of 400 quantitative trait loci (QTL) were selected, accounting for approximately 1% of the total SNPs. In both two simulation scenarios with different numbers of breeds, all chromosomes were divided into blocks using the haplotype definition method proposed in this study with the combined genotype information of breed A and B. The QTL contains two groups of SNPs. Firstly, 300 blocks were randomly selected, and one SNP was chosen as the QTL within each block. The effects of these QTL were sampled from a standard normal distribution, representing no genetic correlation between breeds. Then, in the remaining blocks with SNP counts exceeding 30, 10 blocks were randomly selected, and 10 SNPs were randomly chosen as QTL. The QTL effects are assumed to follow a normal distribution $N(\mathbf{0}, \left[ \begin{matrix} 1 & r_{g} \\ r_{g} & 1 \end{matrix} \right])$. Three levels of genetic correlations (rg = 0.2, 0.5, and 0.8) were considered in the simulations of two breeds, and only one level of rg (0.2) was considered in the simulation of three breeds. The scenario where all 10 blocks had the same rg was named "identical". While in the "uniform", rg were sampled from a uniform distribution U(-1, 1) and then adjusted by adding a constant to set the means at 0.2, 0.5, or 0.8. The true breeding values (TBV) were obtained by multiplying the individual's genotype matrix of QTL(coded as 0, 1, or 2) by the QTL effects. The TBV of each breed was standardized to a standard normal distribution. We set different heritabilities and population means for each breed (Supplemental Table S6). Finally, the population means, TBV and residual effects were summed to obtain the phenotypic values of each individual, which were then standardized to a standard normal distribution for further analysis.

The entire simulation process from genotype to phenotype information was repeated 20 times, and the results were the average of these 20 replications. Only the population mean was considered as fixed effect in WGBP models.

## Real data for model validation

**Pig**: The pig dataset [4] comprised 228 Landrace (LL) and 641 Yorkshire (YY) individuals (total n = 869), and was used to evaluate genomic prediction models for marbling score (MS) and the proportion of fat areas in the image (PFAI). Genotypes were originally generated with the CC1 PorcineSNP50 BeadChip (51,368 SNPs); after removing non-autosomal markers and applying a minor allele frequency (MAF) filter of < 0.01 the working marker set contained 37,304 SNPs for downstream analyses. Phenotypes were obtained as follows: PFAI was derived from digital images of the longissimus dorsi using the MATLAB image-processing pipeline made available by the original study, and MS was scored by three trained panelists on the NPPC 1–10 scale with the median score used as the final MS value.

**Bean**: The bean dataset [1] used in this study consisted of three breeding panels: the climbing bean panel (VEC), the Andean diversity panel (ADP) and the elite Andean breeding panel (VEF). Two traits, 100-seed weight (100SdW) and yield (Yield), were analyzed using BLUEs produced by Keller et al. (2022) as the response variables, thereby minimizing environmental and experimental design effects prior to genomic modeling. The number of lines with phenotypic records per panel ranged from approximately 344 to 587. Genotyping was performed by GBS (ApeKI) and sequence reads were processed through the published pipeline (read trimming, alignment and variant calling); genotype filtering and imputation steps reported in the source include genotype quality and missingness filters, MAF filtering, and imputation with Beagle v5.0 (effective population size Ne = 100) using an available genetic map. The filtered and imputed marker set used for genomic prediction comprised 14,913 SNPs located on 11 chromosomes.

## Derivation of posterior distribution

The model proposed in this study can be expressed in the following matrix form:

$$\begin{aligned} \mathbf{y}_{l}=\mathbf{X}_{l}\boldsymbol{b}+\sum_{i=1}^{s} \sum_{j=1}^{m_{i}} {\boldsymbol{m}_{ijl}\boldsymbol{a}}_{ijl}+\boldsymbol{e}_{l}\boldsymbol{\#}\left( \boldsymbol{1} \right) \end{aligned}$$

where $\boldsymbol{y}_{l}$ is the phenotypes (or BLUEs) vector of breed *l*, ***b*** is the vector of fixed effect with a uniform prior, *s* is the number of blocks across all chromosomes, $m_{i}$ is the number of SNPs in the *i^th^* block, $\boldsymbol{a}_{ijl}$ is the allelic substitution effect of breed *l* at the marker *j* within the *i^th^* block*,* and it follows a multivariate normal distribution with the prior of markers’ effect in the *i^th^* block being $N\left( \boldsymbol{0}, \mathbf{G}_{i} \right)$, and $\mathbf{G}_{i}$ is the (co)variance matrix of all marker effects within the block, with a prior of inverse Wishart distribution $IW\left( df, \mathbf{B}_{i} \right)$ and $\mathbf{B}_{i}=\tilde{h}^{2}\mathbf{P}/[s(df-1)\sum_{j=1}^{m_{i}} 2p_{j}(1-p_{j})]$, $df=4+p$ and *p* is number of breeds, $\tilde{h}^{2}$ is prior of heritability (we used 0.5), **P** is a diagonal matrix with diagonal as phenotypic variance, $p_{j}$ is allele frequency of SNPs *j* in the *i^th^* block, **e** is residual effect vector that follows $N\left( \boldsymbol{0}, {\mathbf{I}\boldsymbol{\otimes}\mathbf{R}}_{0} \right)$, and $\mathbf{R}_{0}\sim IW\left( df,\mathbf{R}_{p} \right)$, $\mathbf{R}_{p}={(1-\tilde{h}}^{2}\boldsymbol{)}\mathbf{P}/(df-1)$. The joint posterior density of all parameters (after augmentation with the missing records), allowing for dependence of the distribution of the residual and additive effects on the corresponding covariance matrices, but assuming prior independence otherwise, is given by:

$$\begin{aligned} p\left( \boldsymbol{b},\boldsymbol{a},\mathbf{G}_{1},\cdots,\mathbf{G}_{s},\mathbf{R}_{0} \right)\propto\\ p\left( \mathbf{y}\mid\boldsymbol{b},\boldsymbol{a},\mathbf{G}_{1},\cdots,\mathbf{G}_{s},\mathbf{R}_{0} \right)p(\boldsymbol{b})\prod_{i=1}^{s} \prod_{j=1}^{m_{i}} \left[ p\left( \boldsymbol{a}_{ij}\mid\mathbf{G}_{i} \right) \right]\prod_{i=1}^{s} \left[ p\left( \mathbf{G}_{i}\mid df,\mathbf{B}_{i} \right) \right] \\ p\left( \mathbf{R}_{0}\mid df,\mathbf{R}_{p} \right) \end{aligned}$$

In the model we proposed, the same traits from different breeds are regarded as different but correlated traits of the same population. Hence, for a given breed, it possesses only one of the traits in the multi-trait model, with the others being marked as missing. Thus, we need to fill in the missing records first. For imputation, missing records were sampled from a normal distribution using a standard data augmentation approach [5], where the conditional distribution of the missing values was derived from the joint posterior of all model parameters:

$$\begin{aligned} \boldsymbol{y}_{k}^{\text{complete }}=\left( \boldsymbol{y}_{k}^{m},y_{k}^{o} \right) \\ p\left( \boldsymbol{y}_{k}^{m}\mid\boldsymbol{y},\mathbf{G},\mathbf{R}_{0} \right)\sim N\left( {\hat{\boldsymbol{y}}}_{k}^{m},\mathbf{V}_{y_{k}}^{m} \right)\sim N\left( E\left( \boldsymbol{y}_{k}^{m}\mid\boldsymbol{b},\boldsymbol{a},\mathbf{R}_{0},\mathbf{G},y_{k}^{o} \right),Var\left( \mathbf{y}_{k}^{m}\mid\boldsymbol{b},\boldsymbol{a},\mathbf{R}_{0},\mathbf{G},y_{k}^{o} \right) \right) \\ E\left( \mathbf{y}_{k}^{m}\mid\boldsymbol{b},\boldsymbol{a},\mathbf{R}_{0},\mathbf{G},y_{k}^{o} \right)=\left[ \begin{aligned} \delta_{1}{\mathbf{z}_{k}\boldsymbol{b}}_{1} \\ \delta_{2}{\mathbf{z}_{k}\boldsymbol{b}}_{2} \\ \vdots\\ \delta_{p}{\mathbf{z}_{k}\boldsymbol{b}}_{p} \end{aligned} \right]+\left[ \begin{matrix} \delta_{1}\boldsymbol{m}_{1}' & 0 & \cdots& 0 \\ 0 & \delta_{2}\boldsymbol{m}_{2}' & \cdots& 0 \\ \vdots& \vdots& & \vdots\\ 0 & 0 & \cdots& \delta_{p}\boldsymbol{m}_{p}^{'} \end{matrix} \right]\left[ \begin{aligned} \boldsymbol{a}_{1}^{*} \\ \boldsymbol{a}_{2}^{*} \\ \vdots\\ \boldsymbol{a}_{p}^{*} \end{aligned} \right]+\mathbf{R}_{0}^{[m,o]}\left( \mathbf{R}_{0}^{[o,o]} \right)^{-1}\boldsymbol{e}^{o} \\ Var\left( \boldsymbol{y}_{k}^{m}\mid\boldsymbol{b},\boldsymbol{a},\mathbf{R}_{0},\mathbf{G},y_{k}^{o} \right)=\mathbf{R}_{0}^{[m,m]}-\mathbf{R}_{0}^{[m,o]}\left( \mathbf{R}_{0}^{[o,o]} \right)^{-1}\mathbf{R}_{0}^{[o,m]} \end{aligned}$$

where $\boldsymbol{y}_{k}^{m}$ is the missing record, and $y_{k}^{o}$ is the observed record for individual *k*, $\boldsymbol{z}_{k}$ and $\boldsymbol{m}_{p}^{'}$is subset of incidence matrix **X** and **M**, respectively.

After augmentation with the missing records, the fully conditional posterior distributions of fixed effects can be derivatized as:

$$\begin{aligned} p(\boldsymbol{b}\mid.)\propto p(\boldsymbol{y}\mid.)&\propto|\mathbf{R}_{0}|^{-1/2}exp\left\{ -\frac{1}{2}\left( \boldsymbol{y}^{*}-\mathbf{X}\boldsymbol{b} \right)^{'}\mathbf{R}^{-1}\left( \boldsymbol{y}^{*}-\mathbf{X}\boldsymbol{b} \right) \right\} \\ &\propto exp\left\{ -\frac{1}{2}\left( \boldsymbol{b}^{'}\mathbf{X}^{'}\mathbf{R}^{-1}\mathbf{X}\boldsymbol{b}-\boldsymbol{b}^{\mathbf{'}}\mathbf{X}^{'}\mathbf{R}^{-1}\boldsymbol{y}^{*}-\boldsymbol{y}^{*'}\mathbf{R}^{-1}\mathbf{X}\boldsymbol{b} \right) \right\} \\ &\propto\left| \left( \mathbf{X}^{'}\mathbf{R}^{-1}\mathbf{X} \right)^{-1} \right|^{1/2}exp\left\{ -\frac{1}{2}\left[ \boldsymbol{b}-\left( \mathbf{X}^{'}\mathbf{R}^{-1}\mathbf{X} \right)^{-1}\mathbf{X}^{'}\mathbf{R}^{-1}\mathbf{y}^{*} \right]^{'}\mathbf{X}^{'}\mathbf{R}^{-1}\mathbf{X}\left[ \boldsymbol{b}-\left( \mathbf{X}^{'}\mathbf{R}^{-1}\mathbf{X} \right)^{-1}\mathbf{X}^{'}\mathbf{R}^{-1}\boldsymbol{y}^{*} \right] \right\} \\ &\propto N\left[ \left( \mathbf{X}^{'}\mathbf{R}^{-1}\mathbf{X} \right)^{-1}\mathbf{X}^{'}\mathbf{R}^{-1}\boldsymbol{y}^{*},\left( \mathbf{X}^{'}\mathbf{R}^{-1}\mathbf{X} \right)^{-1} \right] \\ &=N\left( \frac{1}{n}\mathbf{R}_{0}\mathbf{X}^{'}\mathbf{R}^{-1}\boldsymbol{y}^{*},\frac{1}{n}\mathbf{R}_{0} \right) \\ &=N\left( \frac{1}{n}\mathbf{R}_{0}\mathbf{R}_{0}^{-1}\left( \mathbf{1}_{n}^{'}\bigotimes\mathbf{I}_{p} \right)\boldsymbol{y}^{*},\frac{1}{n}\mathbf{R}_{0} \right) \\ &=N\left( \frac{1}{n}\left( \mathbf{1}_{n}^{'}\bigotimes\mathbf{I}_{p} \right)\boldsymbol{y}^{*},\frac{1}{n}\mathbf{R}_{0} \right) \end{aligned}$$

where $\boldsymbol{y}^{*}$ is the corrected phenotype calculated by $\boldsymbol{y}^{*}=\boldsymbol{y}-(\mathbf{M}\bigotimes\mathbf{I}_{p})\boldsymbol{a}$ and **M** is allele content matrix of all individuals.

The fully conditional posterior distributions of additive effects can be derivatized as:

$$\begin{aligned} p\left( \boldsymbol{a}_{\mathrm{ij}}\mid. \right)&\propto p(\boldsymbol{y}\mid\cdot)p\left( \boldsymbol{a}_{ij}\mid\mathbf{G}_{i} \right) \\ &\propto exp\left\{ -\frac{1}{2}\left( \boldsymbol{y}^{§}-\mathbf{M}_{ij}^{*}\boldsymbol{a}_{ij} \right)^{'}\mathbf{R}^{-1}\left( \boldsymbol{y}^{§}-\mathbf{M}_{ij}^{*}\boldsymbol{a}_{ij} \right) \right\}exp\left\{ -\frac{1}{2}\boldsymbol{a}_{ij}^{'}\mathbf{G}_{i}^{-1}\boldsymbol{a}_{ij} \right\} \\ &\propto exp\left\{ -\frac{1}{2}\left( \boldsymbol{a}_{ij}^{'}\mathbf{M}_{ij}^{*'}\mathbf{R}^{-1}\mathbf{M}_{ij}^{*}\boldsymbol{a}_{ij}-\boldsymbol{y}^{§'}\mathbf{R}^{-1}\mathbf{M}_{ij}^{*}\boldsymbol{a}_{ij}-\boldsymbol{a}_{ij}^{'}\mathbf{M}_{ij}^{*'}\mathbf{R}^{-1}\boldsymbol{y}^{§}+\boldsymbol{a}_{ij}^{'}\mathbf{G}_{i}^{-1}\boldsymbol{a}_{ij} \right) \right\} \\ &\propto N\left[ \left( \mathbf{M}_{ij}^{*'}\mathbf{R}^{-1}\mathbf{M}_{ij}^{*}+\mathbf{G}_{i}^{-1} \right)^{-1}\mathbf{M}_{ij}^{*'}\mathbf{R}^{-1}\boldsymbol{y}^{§},\left( \mathbf{M}_{ij}^{*'}\mathbf{R}^{-1}\mathbf{M}_{ij}^{*}+\mathbf{G}_{i}^{-1} \right)^{-1} \right] \\ &\sim N\left[ \left( \boldsymbol{m}_{ij}^{'}\boldsymbol{m}_{\mathrm{ij}}\mathbf{R}_{0}^{-1}+\mathbf{G}_{i}^{-1} \right)^{-1}\mathbf{R}_{0}^{-1}\left( \boldsymbol{m}_{ij}^{'}\bigotimes\mathbf{I}_{p} \right)\boldsymbol{y}^{§},\left( \boldsymbol{m}_{ij}^{'}\boldsymbol{m}_{ij}\mathbf{R}_{0}^{-1}+\mathbf{G}_{i}^{-1} \right)^{-1} \right] \end{aligned}$$

where $\mathbf{y}^{§}$ is the corrected phenotype calculated as:

$$\begin{aligned} \boldsymbol{y}^{§}&=\boldsymbol{y}-\mathbf{X}\boldsymbol{b}-\sum_{t\neq i}^{s} \mathbf{M}_{t}^{*}\boldsymbol{a}_{t}-\sum_{u\neq j}^{m_{i}} \mathbf{M}_{iu}^{*}\boldsymbol{a}_{iu} \\ &=\boldsymbol{y}^{*}+\mathbf{M}_{ij}^{*}\boldsymbol{a}_{ij}-\mathbf{X}\boldsymbol{b} \\ &=\boldsymbol{y}^{*}+(\mathbf{m}_{ij}\otimes\mathbf{I}_{p})\boldsymbol{a}_{ij}-(\mathbf{1}_{n}\otimes\mathbf{I}_{p})\boldsymbol{b} \end{aligned}$$

Then, the fully conditional posterior distributions of dispersion matrices $\mathbf{G}_{i}$ can be derivatized as:

$$\begin{aligned} p\left( \mathbf{G}_{i}\mid. \right)&\propto\prod_{j=1}^{m_{i}} \left[ p\left( \boldsymbol{a}_{ij}\mid\mathbf{G}_{i} \right) \right]p\left( \mathbf{G}_{i}\mid df, \mathbf{B}_{i} \right) \\ &\propto\prod_{j=1}^{m_{i}} \left[ \left| \mathbf{G}_{i} \right|^{-\frac{1}{2}}exp\left\{ -\frac{1}{2}tr\left( \boldsymbol{a}_{ij}\boldsymbol{a}_{ij}^{'}\mathbf{G}_{i}^{-1} \right) \right\} \right]\left| \mathbf{G}_{i} \right|^{-\left( df+p+1 \right)/2}exp\left\{ -\frac{1}{2}tr\left( \mathbf{B}_{i}\mathbf{G}_{i}^{-1} \right) \right\} \\ &\propto\left| \mathbf{G}_{i} \right|^{-\frac{m_{i}}{2}}exp\left\{ -\frac{1}{2}\sum_{j=1}^{m_{i}} tr\left( \boldsymbol{a}_{ij}\boldsymbol{a}_{ij}^{'}\mathbf{G}_{i}^{-1} \right) \right\}\left| \mathbf{G}_{i} \right|^{-\left( df+p+1 \right)/2}exp\left\{ -\frac{1}{2}tr\left( \mathbf{B}_{i}\mathbf{G}_{i}^{-1} \right) \right\} \\ &\propto\left| \mathbf{G}_{i} \right|^{-\left( df+m_{i}+p+1 \right)/2}exp\left\{ -\frac{1}{2}tr\left[ \left( \sum_{j=1}^{m_{i}} \boldsymbol{a}_{ij}\boldsymbol{a}_{ij}'+\mathbf{B}_{i} \right)\mathbf{G}_{i}^{-1} \right] \right\} \\ &\propto IW\left[ df+m_{i},\left( \sum_{j=1}^{m_{i}} \boldsymbol{a}_{ij}\boldsymbol{a}_{ij}'+\mathbf{B}_{i} \right) \right] \end{aligned}$$

Similarly, the fully conditional posterior distributions of dispersion matrices $\mathbf{R}_{0}$ can be derivatized as:

$$\begin{aligned} p\left( \mathbf{R}_{0}\mid. \right)&\propto p(\boldsymbol{y}|.)p\left( \mathbf{R}\mid df,\mathbf{R}_{p} \right) \\ &\propto{\left| \mathbf{R}_{0}\otimes\mathbf{I}_{n} \right|^{-\frac{1}{2}}exp\left\{ -\frac{1}{2}tr[\boldsymbol{e}\boldsymbol{e}^{'}(\mathbf{R}_{0}\otimes\mathbf{I}_{n})] \right\}\left| \mathbf{R}_{0} \right|}^{-\left( df+p+1 \right)/2}exp\left\{ -\frac{1}{2}tr\left( \mathbf{R}_{p}\mathbf{R}_{0}^{-1} \right) \right\} \\ &\propto\left| \mathbf{R}_{0} \right|^{-\frac{n}{2}}exp\left\{ -\frac{1}{2}tr\left( \boldsymbol{e}\boldsymbol{e}^{'}\mathbf{R}_{0}^{-1} \right) \right\}\left| \mathbf{R}_{0} \right|^{-\left( df+p+1 \right)/2}exp\left\{ -\frac{1}{2}tr\left( \mathbf{R}_{p}\mathbf{R}_{0}^{-1} \right) \right\} \\ &\propto\left| \mathbf{R}_{0} \right|^{-\left( df+n+p+1 \right)/2}exp\left\{ -\frac{1}{2}tr\left[ \left( \sum_{i=1}^{n} \boldsymbol{e}_{k}\boldsymbol{e}_{k}^{'}+\mathbf{R}_{p} \right)\mathbf{R}_{0}^{-1} \right] \right\} \\ &\propto IW\left[ df+n,\left( \sum_{k=1}^{n} \boldsymbol{e}_{k}\boldsymbol{e}_{k}^{'}+\mathbf{R}_{p} \right) \right] \end{aligned}$$

where $\boldsymbol{e}_{k}$ is the residual effect vectors for individual *k*, n is the number of individuals in reference.

## GBLUP models for accuracy comparison

The GBLUP model is widely used in animal and plant breeding, so in this study, two GBLUP models were applied for comparison. The GBLUP model used in MBGP was defined as:

$$\begin{aligned} \boldsymbol{y}=\mathbf{X}\boldsymbol{b}+\mathbf{Z}\boldsymbol{a}+\boldsymbol{e\#}\left( \boldsymbol{1} \right) \end{aligned}$$

where ***y*** is the vector of phenotypes (or BLUEs), ***b*** is the vector of fixed effects, ***a*** is the vector of additive genetic effect, **X** and **Z** are the incidence matrices associating ***b*** and ***a***, ***e*** is the residual effect assumed to follow a normal distribution $N(\boldsymbol{0}, \mathbf{I}\sigma_{e}^{2})$, where **I** is an identity matrix and $\sigma_{e}^{2}$ is the residual effect variance.

This study implemented two extended GBLUP models for MBGP, including STGBLUP and MTGBLUP. In STGBLUP, the different breeds within the reference population are treated as a single breed with a shared genetic background, and single-trait GBLUP is applied for prediction. In this model, ***a*** is supposed to follow a normal distribution $N(\boldsymbol{0}, \mathbf{G}\sigma_{a}^{2})$, where $\sigma_{a}^{2}$ is the variance of additive genetic effect. In contrast, in MTGBLUP, the same trait measured in different breeds is treated as distinct traits within a single breed, and multi-trait GBLUP is used for prediction. In this model, ***a*** is the vector of additive genetic effect that follows a multivariate normal distribution $N(\boldsymbol{0}, \mathbf{G}\bigotimes\mathbf{G}_{0})$, where $\mathbf{G}_{0}$ is the (co)variance matrix of additive genetic effect. Similarly, ***e*** is the vector of random residual effect following a multivariate normal distribution $N(\boldsymbol{0}, \mathbf{I}\bigotimes\mathbf{R}_{0})$, where $\mathbf{R}_{0}$ is the (co)variance matrix of residual effects. In both models, breed was included as a fixed effect. In the pig data analysis, sex was additionally fitted as a fixed effect, while for the bean phenotypes, non-genetic effects had already been removed, and thus only breed was included as a fixed effect. The genomic relationship matrix (**G**) was constructed using the commonly adopted method for genomic prediction [6] implemented in the GMAT software (https://github.com/chaoning/GMAT), based on a merged genotypic dataset that combined multiple breeds.

Two GBLUP models utilized the DMUAI program in the DMU software (https://dmu.ghpc.au.dk/dmu/DMU/) to estimate variance components and GEBVs. It should be noted that, in order to run the MTGBLUP model in DMU, a constraint must be applied to the covariance of residual effects, whereby the off-diagonal elements of $\mathbf{R}_{0}$ are fixed at 0.

# References

[1] Keller B, Ariza-Suarez D, Portilla-Benavides AE, Buendia HF, Aparicio JS, Amongi W, et al. Improving association studies and genomic predictions for climbing beans with data from bush bean populations. Front Plant Sci. 2022;13:830896.

[2] Wientjes Y, Calus M, Duenk P, Bijma P. Required properties for markers used to calculate unbiased estimates of the genetic correlation between populations. Genet Sel Evol. 2018;50:65.

[3] Sargolzaei M, Schenkel FS. QMSim: a large-scale genome simulator for livestock. Bioinformatics. 2009;25:680-1.

[4] Xie L, Qin J, Rao L, Tang X, Cui D, Chen L, et al. Accurate prediction and genome-wide association analysis of digital intramuscular fat content in longissimus muscle of pigs. Animal Genetics. 2021;52(5):633-44.

[5] Gianola D, Fernando RL. A multiple-trait bayesian lasso for genome-enabled analysis and prediction of complex traits. Genetics. 2020;214:305-31.

[6] VanRaden PM. Efficient methods to compute genomic predictions. J Dairy Sci. 2008;91:4414-23.
